# Phase Control Mechanisms in Metasurfaces: From Static Approaches to Active and Space–Time Modulation

**DOI:** 10.3390/s26061781

**Published:** 2026-03-11

**Authors:** Muhammad Haroon, Sun-woong Kim, Dong-You Choi

**Affiliations:** 1 Information and Communication Engineering, Chosun University, Gwangju 61452, Republic of Korea; 2 National Center of Excellence in Software, Chosun University, Gwangju 61452, Republic of Korea

**Keywords:** active/reconfigurable metasurfaces, Huygens metasurfaces, hybrid metasurfaces, metasurfaces, PB phase, phase engineering, propagation phase, resonant metasurfaces, space–time (ST) modulation

## Abstract

Metasurfaces provide a compact and powerful means of tailoring electromagnetic wavefronts through spatially varying phase manipulation. This review presents a unified, mechanism-centered perspective on phase control in metasurfaces, tracing their evolution from static designs to actively reconfigurable and space–time-modulated platforms. Beginning with the theoretical basis of generalized Snell’s law, phase-control strategies are categorized into resonance-based, PB phase, and propagation-phase mechanisms, with emphasis on their underlying physics, bandwidth, efficiency, and polarization characteristics. These static approaches are then extended to active metasurfaces that enable post-fabrication reconfiguration through liquid-crystal tuning, electro-optic, phase-change materials, and mechanical deformation. Beyond quasi-static tuning, space–time modulation is introduced as a distinct paradigm that exploits temporal phase gradients to achieve frequency conversion, nonreciprocity, and waveform synthesis. By organizing diverse implementations around their physical phase-control mechanisms and experimentally reported performance trends, this review provides practical guidance for selecting metasurface architectures across frequency regimes and application requirements.

## 1. Introduction

Controlling the phase of electromagnetic waves with subwavelength resolution underpins beam steering, wavefront shaping, focusing, holography, and polarization manipulation across microwave to optical regimes. Metasurfaces, planar arrays of engineered scatterers, enable abrupt and spatially varying phase discontinuities at deep subwavelength thicknesses, thereby realizing functionalities beyond conventional refractive or reflective optics [1,2,3,4]. Following the generalized laws of reflection and refraction formulated for phase-gradient interfaces [5,6,7], gradient metasurfaces have enabled anomalous reflection and refraction, high-numerical-aperture metalenses, broadband beam steering, and holography across the electromagnetic spectrum [8,9,10,11,12,13].

As metasurface research has expanded rapidly across diverse material platforms, operating frequencies, and device architectures, a wide range of phase-control strategies has emerged. These strategies differ not only in physical mechanism, but also in achievable bandwidth, dispersion behavior, efficiency, polarization selectivity, tunability, and system-level integration constraints. Consequently, a unified perspective that connects static, hybrid, active, and space–time approaches at the level of physical phase-control mechanisms has become increasingly important.

In this spirit, the following sections begin by establishing the core physical mechanisms that enable phase manipulation across metasurface architectures, before progressively extending toward hybrid, tunable, and temporally modulated platforms.

Phase-control mechanisms. Several canonical approaches provide 0–2π (or larger) phase coverage, each characterized by distinct bandwidth, efficiency, polarization response, and fabrication constraints. (i) Resonant (plasmonic or Mie-type) phase control exploits localized resonances to induce strong field confinement and rapid phase variation, enabling compact devices but typically suffering from narrow bandwidths and increased losses at high index contrast [3,14,15]. (ii) PB phase relies on the in-plane rotation of anisotropic meta-atoms, offering broadband and dispersion-robust phase control that is inherently spin-dependent [2,16,17,18,19]. (iii) Propagation-phase manipulation tailors the optical path length through effective-index engineering, typically using high-aspect-ratio dielectric elements to achieve broadband and polarization-insensitive performance [6,8,20,21]. (iv) Huygens metasurfaces achieve full-phase coverage with high efficiency by balancing co-located electric and magnetic dipole responses, enabling reflectionless transmission or refraction under impedance-matched conditions [22,23,24,25,26,27].

Beyond static: hybrid and active control. Hybrid metasurfaces combine multiple phase mechanisms, such as PB and propagation or PB and resonance, to mitigate intrinsic trade-offs related to bandwidth, dispersion, or polarization selectivity [28,29,30,31,32]. Active metasurfaces further extend functionality into real-time beam steering, dynamic holography, and adaptive wavefront control by incorporating electrically, optically, thermally, or mechanically tunable elements, including RF varactors, electro-optic materials, epsilon-near-zero platforms, carrier injection in semiconductors or graphene, phase-change media, and mechanically deformable structures [33,34,35,36,37,38,39].
Toward programmable and space–time metasurfaces. Space-time modulation introduces explicit time dependence into the metasurface response, enabling frequency conversion, harmonic generation, nonreciprocity, Doppler-shift emulation, and frequency-agile beam control, beyond the limits of static phase gradients [40,41,42,43]. Recent demonstrations of space–time-coded RIS, digital coding metasurfaces, and photonic time crystals highlight the growing importance of temporal degrees of freedom from microwave to terahertz regimes [44,45,46].
Scope and contributions. This work presents a mechanism-centered view of metasurface phase control, covering static, hybrid, active, and space–time metasurfaces within a unified physical framework. While many existing surveys focus on a single material platform, frequency band, or tuning modality [47,48,49,50,51], the emphasis here is placed on the underlying physical principles governing phase manipulation and the associated trade-offs in bandwidth, efficiency, tunability, and loss across diverse implementations. Quantitative performance trends are discussed contextually within each mechanism class, with detailed numerical ranges provided in the Supplementary Information to ensure traceability and to avoid over-interpretation of heterogeneous metrics. Table 1 summarizes the positioning of this review relative to prior metasurface surveys in terms of mechanism coverage, theoretical depth, and inclusion of active and space–time modulation.

**Figure 1:**
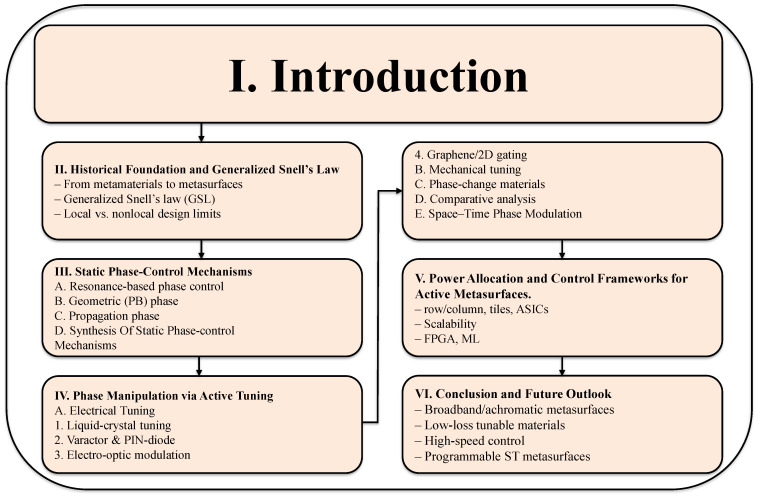
Structure and organization of this review paper, outlining the progression from fundamental phase–control principles to hybrid, active, and space–time modulation mechanisms in metasurfaces.

The remainder of this paper is organized as follows. Figure 1 illustrates the overall structure of the review. Section 2 introduces the theoretical foundations of phase discontinuities and wavefront control. Section 3 surveys static phase-control mechanisms. Section 4 examines active metasurfaces. Section 5 presents power allocation and control frameworks for active metasurfaces. Section 6 discusses practical application challenges across different metasurface mechanisms. Section 7 concludes the review and outlines future research directions.

## 2. Historical Foundations

The conceptual origins of metamaterials and metasurfaces trace back to Veselago’s theoretical study of media with simultaneously negative permittivity and permeability [53]. Although experimental realizations emerged decades later using bulk metamaterials [54,55,56], challenges such as ohmic loss, fabrication complexity, and limited scalability [57,58,59] motivated the transition toward ultrathin metasurfaces capable of wavefront control through engineered surface phase profiles [1,60]. This shift from volumetric to planar architectures marked a conceptual change in how phase accumulation and wave manipulation could be achieved. A broader comparison of conventional optical elements, bulk metamaterials, and metasurfaces is summarized in Table 2. The table contrasts their dominant phase mechanisms, thickness, tunability, and practical performance considerations, highlighting how metasurfaces emerged as a lightweight and versatile platform for wavefront engineering.

**Figure 2:**
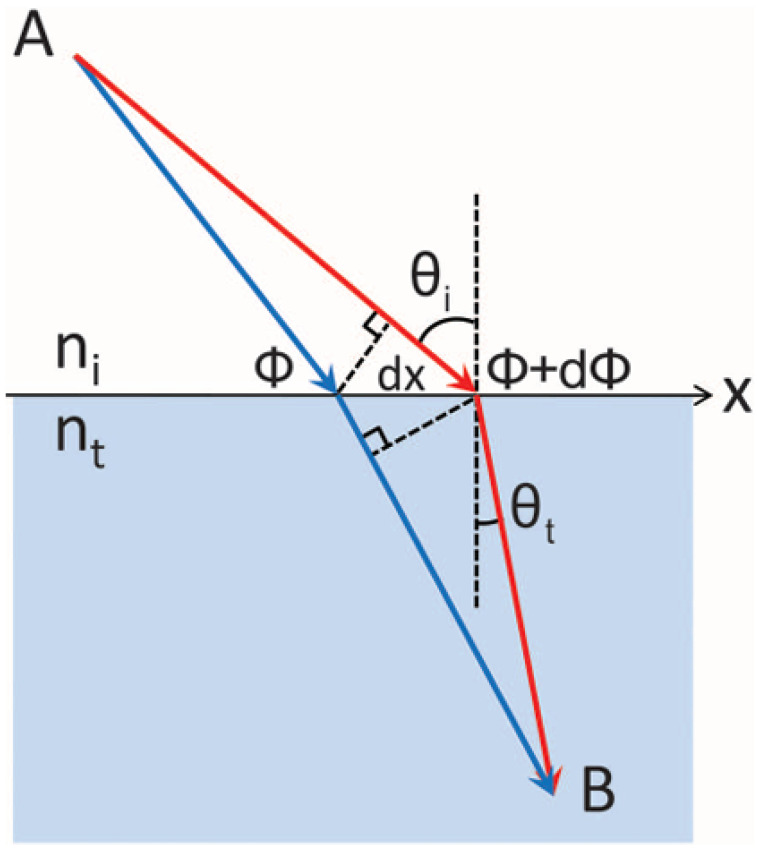
Generalized Snell’s law. A spatial phase gradient dΦ/dx imposed at an interface deflects the transmitted beam from an incident angle $\theta_{i}$ to a transmitted angle $\theta_{t}$. The blue and red rays represent two infinitesimally close optical paths crossing the interface, where Φ and Φ+dΦ denote the phase discontinuities introduced at the boundary and dx is the separation between the two crossing points along the interface (adapted from [5]).

Within this historical evolution, a central question concerns how a thin interface can redirect electromagnetic waves in a controllable manner. As illustrated in Figure 2, generalized Snell’s law (GSL) provides an intuitive link between spatially varying interfacial phases and macroscopic beam steering. Yu et al. [5] showed that an abrupt phase discontinuity Φ(x) modifies the tangential momentum at an interface, yielding

(1) $$n_{t}\sin\theta_{t}-n_{i}\sin\theta_{i}=\frac{\lambda_{0}}{2\pi }\frac{d\Phi }{dx},$$

with an analogous expression for anomalous reflection. This formulation extends classical Snell’s law by treating the metasurface as a locally periodic interface whose unit cells impose a prescribed phase gradient.

While this formulation provides a clear physical picture of beam deflection, its applicability is inherently tied to the assumptions used in its derivation. It is important to emphasize that these generalized laws were derived under specific assumptions, including weak inter-element coupling, dipolar unit-cell responses, and slowly varying or locally periodic phase profiles. Consequently, GSL functions primarily as a first-order design guideline rather than a universally predictive model for metasurface performance. In practical implementations, full-wave coupling effects, unit-cell dispersion, finite phase discretization, and material losses can introduce deviations from the beam directions predicted by GSL [61,62].

These limitations have motivated alternative design paradigms that relax the local periodicity assumption underlying GSL. Recent studies demonstrate that nonlocal or multi-cell-coupled metasurfaces can exceed the performance of locally designed GSL-based implementations, particularly for large-angle steering. By explicitly accounting for inter-element coupling, such nonlocal designs suppress spurious diffraction channels and improve overall efficiency [62]. These results underscore that optimal metasurface performance often requires moving beyond strictly local phase prescriptions.

From a practical perspective, phase-gradient relations must therefore be viewed in conjunction with achievable phase coverage. Finally, for efficient wavefront control, a complete 0–2π phase coverage is typically required, which can be achieved through resonant engineering or geometric tuning of the meta-atoms [63]. Taken together, generalized Snell’s law provides an intuitive conceptual framework for phase-gradient metasurfaces, while actual device performance is ultimately governed by full-wave interactions, material constraints, and, in many cases, nonlocal or multi-parameter optimization strategies.

**Table 2:** Comparison of conventional optics, metamaterials, and metasurfaces.

System	Phase Mechanism	Thick	Bandwidth	Tunability	Scattering Control	Notes	References
Conventional Optics	Propagation phase	Thick	Broadband	None	No	Bulky refractive elements	[64,65]
Metamaterials	Resonant (SRR/wire)	Thick	Narrow	Limited	Moderate	Lossy; hard to scale to optical freq.	[54,55,57,66,67]
Metasurfaces	Resonant/PB/Propagation	Ultrathin	Mechanism- dependent	High (active)	Excellent	Holography, beam steering, STM	[5,51,68,69]

## 3. Static Phase Control Mechanisms

Static phase control mechanisms refer to approaches where the phase profile of a metasurface is permanently defined by its geometry or material properties, without external tuning. These mechanisms rely on fixed structural parameters—such as resonator dimensions, orientation, or thickness—to impart a spatially varying phase shift across the surface, enabling functionalities like beam steering, focusing, and polarization conversion under static conditions. As the earliest and most widely explored class of metasurfaces, static mechanisms provide the physical foundation upon which more advanced active and space–time-modulated approaches are later developed.

Within static metasurfaces, phase control can be realized through several distinct physical mechanisms. The following subsections examine these mechanisms in turn, beginning with resonance-driven phase modulation, which historically represents the first widely adopted approach.

### 3.1. Resonance-Based Phase Control

**Figure 3:**
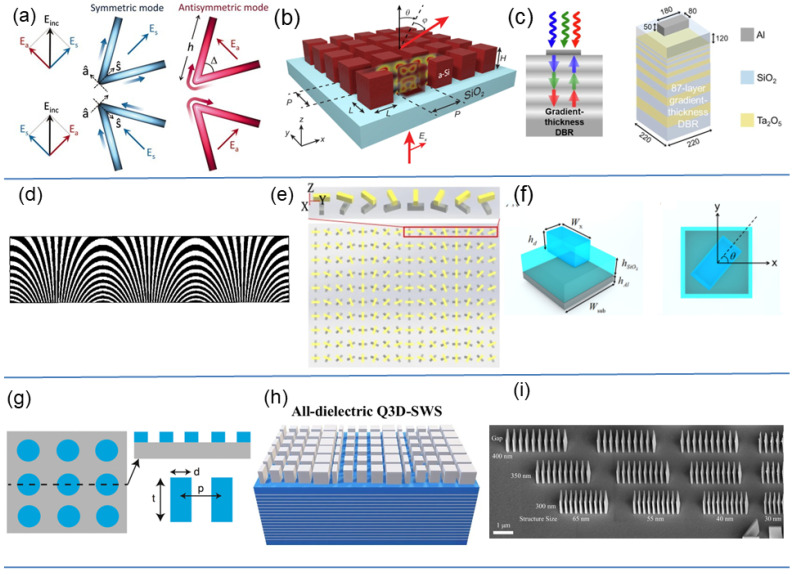
(**a**) Plasmonic V-antenna: electric-dipole (symmetric) and gap-like magnetic (antisymmetric) modes used for phase gradients [5]. (**b**) All-dielectric unit cell: a-Si nanoblocks on SiO_2_ for resonance-only phase control (adopted from [70]). (**c**) Microcavity-assisted unit cell: anisotropic meta-atom with a gradient-thickness DBR providing multiple high-Q channels (adopted from [31]). (**d**) Space-variant grating showing PB phase from in-plane rotation [18]. (**e**) Practical PB encoder: rotated nanofins with $\phi_{\mathrm{PB}}=\pm 2\theta$ (spin-dependent) (adopted from [71]). (**f**) Unit-cell geometry and orientation (a-Si on SiO_2_ over Al); top view shows the rotation angle θ [72]. (**g**) Dielectric metalens unit cell consisting of cylindrical nano-posts with diameter d, lattice periodicity p, and thickness t (adopted from [73]). (**h**) Quasi-3D subwavelength structure for low-loss, high-efficiency phase control [74]. (**i**) Tilt-view SEM of dielectric grating metasurface illustrating propagation-phase control via duty-cycle variation (adopted from [75]).

Figure 3a–c illustrate three representative resonant platforms—plasmonic V-antennas, dielectric Mie resonators, and microcavity-assisted meta-atoms—which serve as visual examples throughout this subsection.

Resonant metasurfaces achieve phase modulation by exploiting the interaction of incident light with subwavelength resonant elements. The basic mechanism is captured by the Lorentz oscillator model, where a bound electron of mass *m* and charge *q* responds to a harmonic driving field as

(2) $$m\ddot{x}+m\gamma \dot{x}+m\omega_{0}^{2}x=qE_{0}e^{-i\omega t},$$

a formulation introduced in classical electrodynamics and widely used to describe material dispersion [76]. The associated polarizability,

(3) $$\alpha \left(\omega \right)=\frac{q^{2}/m}{\omega_{0}^{2}-\omega^{2}-i\gamma \omega },$$

produces a phase response

(4) $$\phi \left(\omega \right)=\arctan\left(\frac{\gamma \omega }{\omega_{0}^{2}-\omega^{2}}\right).$$

Such dipolar polarizability models form the analytical foundation for describing the scattering and phase response of subwavelength resonant inclusions, as developed in the classical metasurface modeling framework of Tretyakov [77].

(1) Basic Principle. This response means that a single resonance provides a 0–π phase shift: the induced dipole is in-phase below resonance, lags by $90^{\circ }$ at resonance, and approaches a $180^{\circ }$ lag above it. As a historical note, this resonance-driven phase evolution is closely connected to the artificial magnetic responses first formalized by Pendry et al. [78], which laid the foundation for metamaterial and metasurface resonators. Achieving full 2π phase therefore requires multiple resonances or balanced electric–magnetic modes.

(2) Phase-Control Method. Because the resonant phase in Equation (3) varies sharply around $\omega_{0}$, tuning the geometry of the meta-atom provides a direct means to shift the resonance and thereby assign a desired scattering phase. In plasmonic metasurfaces, this principle was first demonstrated using V-shaped dipole antennas, where arm-length detuning modifies the dipolar resonance and produces a monotonic phase gradient across the array [5]. Subsequent plasmonic implementations have also adopted gap-surface-plasmon (GSP) resonators in metal–insulator–metal configurations, where varying the strip or nanobrick width, length, or gap shifts the confined GSP mode and enables near-complete reflective phase coverage for beam steering and focusing [79,80]. Dielectric metasurfaces operate on the same resonance-tuning principle, with Mie or Fano resonances controlled by adjusting the high-index resonator dimensions, while microcavity-assisted cells extend this approach by exploiting vertically confined Fabry–Pérot modes to realize spectrally selective phase responses.

(3) Limitations. Resonant phase control is inherently narrowband. Plasmonic elements suffer from Ohmic loss and amplitude nonuniformity, while dielectric high-*Q* resonators improve efficiency at the cost of increased sensitivity to fabrication tolerances and angle of incidence. A single resonance provides at most π phase shift, making full 2π control dependent on dual-resonant or hybrid designs.

(4) Practical Implementations. Plasmonic resonators remain useful for compact, visible-range beam steering and holography. All-dielectric Mie resonators enable high-efficiency metalenses and broadband beam shaping. Microcavity-assisted designs add spectral selectivity, supporting multiwavelength holography. Balanced electric and magnetic modes lead naturally to Huygens metasurfaces [22,81], linking this subsection to the next.

Overall, Figure 3a–c captures the evolution from plasmonic to dielectric and cavity-enhanced resonators, all governed by common resonance physics that defines the fundamental efficiency–bandwidth–phase trade-offs inherent to this class of metasurface. For a comprehensive analysis of the physical mechanisms enabling full 2π phase coverage in dielectric resonators and their associated transmission enhancement effects, readers are referred to Ref. [82].

### 3.2. Geometric Phase (Pancharatnam–Berry Phase)

The geometric (PB) phase stems from the trajectory of a polarization state on the Poincaré sphere, not from optical path length [16,17]. In metasurfaces, an anisotropic nanoelement rotated by an in-plane angle θ imposes a PB phase $\phi_{\mathrm{PB}}=\pm 2\theta$ on the cross-polarized component of circularly polarized (CP) light; the sign follows the incident spin [18]. A compact way to see this is via the Jones description of a rotated, birefringent cell:

(5) $$t\left(\theta \right)=R\left(-\theta \right)\;\mathrm{diag}\left(t_{u},t_{v}\right)\;R\left(\theta \right),$$

where R(θ) is the 2×2 rotation matrix

$$R\left(\theta \right)=\left(\begin{array}{cc} \cos\theta & -\sin\theta \\ \sin\theta & \cos\theta \end{array}\right),$$

which transforms the electric field between the laboratory (x,y) basis and the principal-axis basis of the anisotropic nanoelement. The quantities $t_{u}$ and $t_{v}$ denote the complex transmission coefficients for linearly polarized light aligned with the two orthogonal principal axes (u,v) of the nanoantenna, accounting for both amplitude and phase response along each axis. For a circularly polarized incident field $E_{R/L}^{\mathrm{in}}=\left(\hat{x}\pm i\hat{y}\right)/\sqrt{2}$, the transmitted field consists of two components: a co-helicity term proportional to $(t_{u}+t_{v})/2$, and a cross-helicity term proportional to $(t_{u}-t_{v})/2$ carrying an additional phase factor $e^{\pm i2\theta }$, where the sign corresponds to the incident spin. Thus, by rotating otherwise identical cells from θ=0 to π, the cross-polarized wave acquires a programmable 0→2π phase purely from orientation. Because $\phi_{\mathrm{PB}}$ depends on angle rather than thickness, the phase is achromatic in principle and, with low-loss dielectrics, can be imparted with high efficiency [4,52].

This orientation-to-phase mapping provides a fundamentally different route to wavefront control than resonance- or propagation-based approaches. The key features and trade-offs of PB-phase metasurfaces can be organized as follows.

(1) Basic Principle. PB-phase metasurfaces convert geometric rotation into phase. Unlike resonance-driven designs, the phase does not depend on optical path or thickness but on the orientation of an anisotropic scatterer, as visualized in Figure 3d–f.

(2) Phase-Control Method. Rotation of identical nanofins or gratings assigns a local phase of ±2θ. Achieving high polarization conversion requires the nanoelement to operate close to the half-wave-plate condition (i.e., $|t_{u}\left|\approx \right|t_{v}|$ with a π phase difference); deviations from this retardance reduce efficiency. While the phase itself is geometric and achromatic, the conversion amplitude depends on material birefringence and therefore remains frequency-dependent.

This baseline picture clarifies both the opportunity and the practical constraints. High conversion to the cross-polarized channel requires local retardance near π; deviations reduce efficiency. The phase is spin-locked (±2θ for opposite CP), so independent control of multiple polarization channels typically needs deliberate multiplexing [83,84]. Moreover, while the PB phase is geometric, the conversion efficiency and transmission remain dispersive because the effective birefringence that sets the retardance varies with frequency; oblique incidence and fabrication tolerances further perturb the target retardance.

(3) Limitations. PB devices are inherently spin-dependent, provide phase only in the converted (cross-polarized) channel, and require maintaining a half-wave condition across the aperture. Efficiency is sensitive to wavelength and fabrication tolerances, and dispersion in birefringence limits the practical achromaticity of PB elements. In this context, representative experimental ranges for phase coverage, efficiency, and bandwidth reported for PB-phase metasurfaces across different material platforms are summarized in Table 3.

Subsequent developments address these issues while preserving the simplicity of angle-to-phase encoding. First, replacing metals with high-index dielectrics suppresses ohmic loss and provides strong-form birefringence, making the half-wave condition achievable with high transmission and thus improving PB conversion efficiency [85]. Second, polarization multiplexing—via interleaved supercells or stacked layers—yields independent phase maps for different spin or linear bases; controlled interlayer twists in bilayer PB stacks tailor symmetry and deterministically tune deflection angles or vortex charge [71]. Third, dispersion engineering shapes the amplitude response while leaving the PB law intact, enabling achromatic implementations [9,75,86]. For narrowband applications, PB rotation can be embedded within high-*Q* resonant structures [87]. Finally, tunability follows naturally from controlling retardance: electro-optic liquid-crystal overlays modulate PB conversion without re-patterning the optic axis [88,89], and magneto-optical bias provides selective spin routing [72,90].

(4) Practical Implementations. Dielectric PB metasurfaces now enable high-efficiency holography, beam shaping, achromatic metalenses, and polarization multiplexing [9,69,91]. Hybrid PB–resonant platforms use rotation for spatial control while resonant elements supply spectral selectivity for multiwavelength functionality [31,87].

Compared to resonance-based phase control, PB-phase metasurfaces offer broadband, low-loss, and structurally simpler 0–2π phase encoding. Their remaining constraints—spin selectivity, retardance accuracy, and dispersion in conversion efficiency—are well understood and routinely mitigated in modern dielectric platforms. A transition to propagation-phase metasurfaces follows naturally, since both rely on non-resonant phase accumulation but differ in how the phase is engineered across the aperture. A more detailed semi-analytical treatment of nanofin-based PB phase metasurfaces, including explicit modeling of polarization conversion efficiency, is provided in Ref. [92].

### 3.3. Propagation Phase Mechanism

Propagation–phase control provides a broadband, nonresonant route to wavefront shaping. Unlike resonance-based or PB approaches, the phase is accumulated as an optical path delay inside each meta-atom,

(6) $$\phi =k\;n_{\mathrm{eff}}\;h$$

with wave number *k*, effective index $n_{\mathrm{eff}}$, and height *h*. Because ϕ varies continuously with geometry and is not tied to a narrow resonance, these metasurfaces can be efficient and relatively polarization-insensitive over wide bands, which underpins their use in lenses and beam shapers [93,94]. A detailed theoretical treatment is given in [52]. In practice, the waveguide-like nanofin building block used for metalensing—the TiO_2_ unit in Figure 3g—is the canonical example of this mechanism.

(1) Basic Principle. Propagation-phase metasurfaces control phase by engineering optical path length rather than by using resonances or polarization rotation. Each meta-atom behaves as a short waveguide whose effective index and height determine the accumulated phase shift. This mechanism is illustrated by the nanopillar and nanofin geometries in Figure 3g,h.

(2) Phase-Control Method. Changing the pillar width, height, or duty cycle tunes $n_{\mathrm{eff}}$ and therefore the accumulated phase. Because this tuning is continuous, full 2π phase coverage is achieved without relying on a resonance condition. This approach is robust, broadband, and largely insensitive to input polarization, which explains its dominance in metalens design.

Early demonstrations established both the building blocks and practical limits of this approach. High-index dielectric nanopillars and nanofins enabled gradient metasurfaces for steering and focusing [73,74]. TiO_2_ nanofins subsequently provided full 2π phase coverage with high transmission and subwavelength sampling [95,96], leading to visible metalenses with diffraction-limited performance [9]. These path-length elements correspond to the concept shown in Figure 3g. Two key bottlenecks were quickly identified: first, tall and high-aspect-ratio features challenge fabrication uniformity; second, material and waveguide dispersion introduce chromatic focal shifts, as conceptually illustrated by the achromatic design strategy in Figure 3h.

Recent work has focused on mitigating these bottlenecks. Advances in nanoscale fabrication have narrowed the gap between design and experiment, pushing measured focusing efficiencies toward theoretical limits [97]. To address chromatic dispersion, several architectures now engineer both phase and group delay. Dispersion-matched multilayers and compensation layers integrated with nanopillars enable achromatic focusing across the visible spectrum [86,98]. In parallel, design strategies based on meta-atom families distribute dispersion across the aperture to achieve broadband achromatic response [99]. Fabrication views such as duty-cycle sweeps highlight how fine control of feature width and fill factor governs the effective index and accumulated phase. Integration advances further reduce system-level barriers, as demonstrated by high-aspect-ratio Si_3_N_4_ metalenses co-fabricated on CMOS imagers with focusing efficiencies exceeding 80% over 460–650 nm and full-color imaging capability [75]. Beyond library-based layouts, inverse-designed slab-waveguide metasurfaces have demonstrated multiwavelength focusing with improved sidelobe suppression [100].

(3) Limitations. Propagation-phase metasurfaces require tall dielectric features that can be difficult to fabricate with high uniformity. Although the phase response is continuous, it is susceptible to chromatic dispersion, leading to focal-length shifts under broadband illumination. Mitigating these effects typically necessitates multilayer stacks, meta-atom families, or computational inverse design [101,102]. Against this background, representative experimental ranges for phase coverage, efficiency, and bandwidth reported for propagation-phase metasurfaces across different platforms are summarized in Table 3.

Taken together with the broader body of experimental and theoretical studies, these results map a coherent progression: early nanopillar and nanofin elements (as in Figure 3g) establish efficient broadband phase control; improved fabrication enhances realized efficiency; dispersion-engineered stacks and meta-atom families (Figure 3h) mitigate chromatic aberrations; and inverse or waveguide-coupled designs [103] expand functionality for complex apertures. Recent reviews synthesize these trade-offs and outline pathways toward large-area, manufacturable achromatic metalenses [104,105]. Finally, quasi three-dimensional stacks that co-design vertical cavities or films with a lateral metasurface phase have achieved near-unity simulated transmission and wide field-of-view beam steering [74].

(4) Practical Implementations. Propagation-phase metasurfaces enable high-efficiency, low-loss metalenses and beam shapers across the visible and near infrared [6,8,21,68]. Their broadband nature has made them central to achromatic focusing [9,10,86,98,99], computational imaging [75,100], and compact optical systems [6,68,93]. Propagation-phase metasurfaces therefore provide a broadband, low-loss foundation that can also be combined with PB or resonant elements when additional polarization or spectral selectivity is required. For readers interested in rigorous theoretical descriptions of effective-index extraction, invariant optical properties, and semi-analytical modeling of dielectric meta-atoms, detailed treatments can be found in Refs. [106,107].

### 3.4. Detour Phase Metasurfaces

Beyond effective-index-based path-length accumulation, a related but conceptually distinct static mechanism is the so-called detour phase, in which phase modulation is encoded through controlled lateral displacement of meta-atoms within a periodic lattice. Originating from classical diffraction-grating theory, a spatial shift Δx introduces a diffraction-order-dependent phase offset proportional to 2πmΔx/P, where *P* is the lattice period and *m* the diffraction order. Unlike propagation phase, which accumulates optical delay inside each meta-atom, detour phase arises purely from positional translation relative to the lattice reference frame. Although less widely adopted in high-NA metalenses, detour-phase metasurfaces provide a valid and structurally simple route to wavefront engineering and have been highlighted in recent comprehensive surveys of advanced metasurface design [108,109,110].

Complementing these analytically parameterized static phase mechanisms, freeform metasurfaces designed via topology optimization or inverse-design techniques have also demonstrated full phase control without relying on parameterized meta-atom libraries. In such approaches, the dielectric distribution within each unit cell is directly optimized using full-wave electromagnetic solvers, enabling non-intuitive geometries that extend beyond simple propagation- or resonance-based descriptors [111].

**Table 3:** Mechanism-level summary of representative experimental performance ranges for static phase-control approaches. The values reported here indicate typical experimentally demonstrated ranges across different frequency bands and application scenarios. Because efficiency definitions (e.g., reflection efficiency, transmission efficiency, focusing efficiency, or aperture efficiency) and bandwidth extraction methods vary between studies, band-resolved, per-paper quantitative comparisons with explicitly defined metrics are provided in Supplementary Table S1. The corresponding references are listed in the Supplementary Materials.

Mechanism	Phase Range	Efficiency	3-dB BW	Notes	References
Resonant (plasmonic)	near 0–2π (design λ)	∼40–80% (reflection)	few % to ∼20%	Resonance-dispersive phase control via plasmonic dipole, gap-surface-plasmon (GSP), or BIC-assisted modes; compact footprint but narrowband and loss-limited.	[13,14,104,106,107]
Propagation (dielectric)	full 0–2π	∼50–90% (aperture/focusing)	∼50–65% (microwave GRIN) broadband (opt.)	Nonresonant effective-index or waveguide-like phase accumulation; broadband and low-loss, with chromatic focal shift in optical metalenses.	[108,109,110,111,112]
PB/Geometric phase	full 0–2π (spin-dep.)	∼13–86% (platform-dep.)	intrinsically broadband	Orientation-induced PB phase; broadband phase response but polarization-locked; efficiency set by retardance accuracy and material loss.	[9,85,91,113]
Huygens (dielectric)	full 0–2π	∼82–>90% (transmission)	∼40 nm (opt.) ∼15–20% (microwave)	Balanced electric and magnetic dipole resonances enabling impedance matching and high-efficiency transmission or reflection.	[114,115,116]
PB + Propagation (hybrid)	full 0–2π	∼20–30% (plasmonic) to >90% (nonres. μw)	>40% (reported)	PB phase provides spin selectivity while propagation phase provides continuous tuning; performance strongly platform-dependent.	[89,117,118,119,120]
PB + Resonance	full 0–2π	∼25–40%	∼25% to ∼50%	PB phase combined with resonant dispersion (e.g., achromatic or multi-channel operation) at the cost of bandwidth and efficiency.	[10,121]
Multi-resonant hybrid	full 0–2π	∼50–85%	multi-peak /band-selective	Multiple resonances per unit cell enabling multi-band focusing/steering and multiplexed responses.	[31,122]

### 3.5. Synthesis of Static Phase-Control Mechanisms

The static phase-control mechanisms discussed above, resonant, PB, propagation-based, Huygens, and their hybrids, span a broad design space defined by distinct physical origins of phase accumulation. Table 3 consolidates representative experimental ranges for phase coverage, efficiency, and bandwidth reported across these approaches. Rather than implying direct performance ranking, the table highlights how intrinsic trade-offs emerge from the underlying mechanism itself: resonant designs emphasize compactness at the cost of bandwidth, propagation-phase metasurfaces favor broadband and low-loss operation with dispersion considerations, PB-phase approaches offer achromatic geometric control with polarization constraints, and Huygens and hybrid platforms balance efficiency and spectral response through mode engineering.

This synthesis underscores that static metasurface performance must be interpreted within the context of the governing phase mechanism and target application. These static strategies form the foundation upon which active and space–time-modulated metasurfaces build additional degrees of freedom, as discussed in the following sections.

## 4. Phase Manipulation via Active Tuning

Active tuning reprograms a metasurface after fabrication by modulating each meta-atom’s effective impedance or birefringence using an external stimulus, such as electrical biasing, optical pumping, thermal switching, or mechanical strain. In practice, this enables dynamic control over the transmitted or reflected phase and, in many implementations, the amplitude as well, allowing real-time beam steering, adaptive focusing, and waveform manipulation without altering the physical layout of the metasurface.

In contrast to the static and hybrid phase-control mechanisms summarized in Table 3, actively tunable metasurfaces introduce additional degrees of freedom associated with temporal reconfigurability, at the expense of increased material, electrical, or system-level complexity. Depending on the tuning mechanism, active platforms exhibit different trade-offs among achievable phase range, modulation speed, optical efficiency, and power consumption. For example, phase-change-material-based metasurfaces offer non-volatile phase states with negligible power required to hold a programmed configuration, whereas electrically driven approaches enable faster update rates but typically incur added conductor, biasing, or insertion losses.

To contextualize these trade-offs relative to the static and hybrid mechanisms discussed earlier, Table 3 provides a quantitative reference point for phase range, bandwidth, and efficiency, against which the benefits and limitations of active tuning can be assessed. Building on this comparison, the following subsections examine the principal active tuning strategies—including liquid-crystal, electro-optic, graphene and other two-dimensional materials, mechanical deformation, phase-change materials, and space–time modulation—focusing on their underlying physical mechanisms and system-level performance characteristics.

### 4.1. Electrical Tuning Mechanisms in Metasurfaces

Electrical tuning provides a powerful and versatile route for dynamic phase modulation in metasurfaces. By applying external bias voltages to individual meta-atoms or by integrating electrically responsive materials into the unit-cell design, the effective impedance, refractive index, or anisotropy of each element can be reconfigured in real time. As a result, the local phase response of the metasurface can be dynamically programmed with high spatial and temporal control.

In the following, electrical tuning mechanisms are categorized according to their dominant physical operation. These include varactor- and PIN-diode-loaded metasurfaces commonly used at microwave and millimeter-wave frequencies, electro-optic platforms based on materials such as LiNbO_3_ and BaTiO_3_, carrier-density modulation in epsilon-near-zero (ENZ) media, and electrically gated graphene and other two-dimensional materials. Each approach offers a distinct balance between phase range, tuning speed, loss, integration complexity, and scalability, which is discussed in detail in the subsections below.

#### 4.1.1. Liquid Crystal Tuning

The operation of an LC-tuned metasurface hinges on the sensitive dependence of meta-atom resonances on the local refractive index. A typical device configuration consists of a metasurface fabricated on a transparent substrate, enclosed within an LC cell with transparent electrodes (such as indium tin oxide, ITO). In the absence of an applied electric field (OFF state), the LC molecules maintain a predefined uniform alignment (for example, planar or homogeneous), dictated by alignment layers. In this state, the meta-atoms are surrounded by a medium with an effective refractive index $n_{\mathrm{LC},\mathrm{OFF}}$, typically equal to the ordinary index $n_{o}$ for normally incident light.

Upon application of a voltage above a threshold (ON state), LC molecules with positive dielectric anisotropy (Δϵ>0) reorient toward the electric field (often homeotropic). This reconfiguration alters the effective refractive index experienced by the metasurface mode to $n_{\mathrm{LC},\mathrm{ON}}\approx n_{e}$ for suitable polarizations. The resulting change

(7) $$\Delta n_{\mathrm{LC}}=n_{\mathrm{LC},\mathrm{ON}}-n_{\mathrm{LC},\mathrm{OFF}}$$

shifts the resonance and phase response of the meta-atoms [116]. A convenient phase model is

(8) $$\phi \left(\lambda ,V\right)\approx \frac{2\pi }{\lambda }\;L_{\mathrm{eff}}\;n_{\mathrm{eff}}\left(V\right),$$

which emphasizes that voltage tuning directly modulates the effective refractive index and therefore the imparted phase.

**Figure 4:**
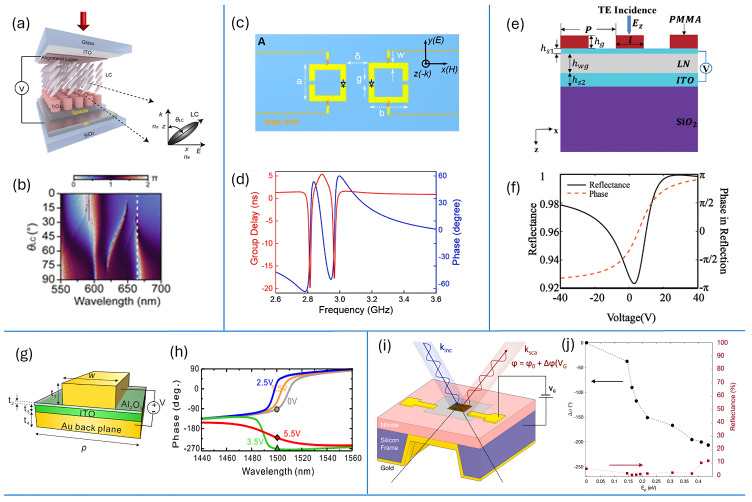
Representative platforms for dynamic phase control in metasurfaces. (**a**,**b**) Liquid-crystal (LC) reflective metasurface: (**a**) practical cell with transparent electrodes; (**b**) reflectance/phase colormap versus wavelength and LC tilt showing near-continuous phase control (adopted from [125]). (**c**,**d**) Diode-tuned microwave metasurface: (**c**) asymmetric split-ring resonator (ASRR) pair with bias lines; (**d**) simulated group delay (red, left axis) and reflection phase (blue, right axis) near the resonances used for phase switching (adopted from [113]). (**e**,**f**) ITO-LiNbO_3_ guided-mode-resonant (GMR) device: (**e**) gated stack cross-section; (**f**) measured reflectance (solid, left axis) and reflection phase (dashed, right axis) versus gate voltage, evidencing electrical phase modulation (adopted from [114]). (**g**,**h**) ITO MOS (ENZ) metasurface: (**g**) metal/Al_2_O_3_/ITO/Au back-plane unit cell; (**h**) phase spectra at several gate biases showing bias-dependent dispersion (adopted from [128]). (**i**,**j**) Graphene-gated mid-IR metasurface: (**i**) device schematic with graphene over a resonant cavity; (**j**) measured phase shift (red, right axis) and reflectance (black, left axis) versus electrostatic tuning, demonstrating $>230^{\circ }$ phase modulation (adopted from [115]). Abbreviations: LC = liquid crystal; ASRR = asymmetric split-ring resonator; GMR = guided-mode resonance; MOS = metal–oxide–semiconductor; ENZ = epsilon-near-zero.

LC tuning offers a large tuning range with low static power consumption. With modern high-birefringence mixtures ($\Delta n=n_{e}-n_{o}≳0.3$) [118,123], resonance shifts of tens of nanometers and phase swings exceeding π can be achieved in well-confined modes [89]. Prominent demonstrations include tunable metalenses with voltage-controlled focal length and polarization-multiplexed focusing [119,120], beam steering over multi-degree angles using LC-infiltrated all-dielectric metasurfaces [116,121], and dynamic holography with switchable or video-rate metaholograms [122,124]. Recently, LC-assisted phase-only metasurface spatial light modulators have achieved near-2π continuous phase control with high efficiency [125]. As illustrated in Figure 4a,b, electrically rotating the nematic LC sweeps the metasurface reflection phase nearly continuously to 2π at λ≈665nm while maintaining high reflectance.

Representative ranges for phase tunability, response time, and insertion loss reported for LC-tuned metasurfaces across different implementations are summarized in Table 4.

The principal limitation of LC tuning is response time. Nematic relaxation typically scales as

(9) $$\tau_{\mathrm{off}}∼\frac{\gamma_{1}d^{2}}{\pi^{2}K_{11}},$$

highlighting a trade-off between switching speed and cell thickness [126,127], where $\gamma_{1}$ is the rotational viscosity, *d* is the LC layer thickness, and $K_{11}$ denotes the splay elastic constant of the nematic liquid crystal that governs the restoring torque of director distortions. As a result, LC-based metasurfaces generally operate at millisecond time scales, which are sufficient for dynamic holography and spatial light modulator-type applications but inadequate for high-speed beam steering or communication systems [89,116,122,125]. Dual-frequency or polymer-stabilized LC formulations can accelerate response at the cost of increased drive complexity or reduced tuning range. Additional challenges include maintaining uniform director alignment in complex near-field environments and minimizing scattering from director nonuniformities. Future directions focus on high-Δn, low-viscosity materials [118], compact LC cells, hybrid LC–electro-optic architectures, and tighter co-integration with electronic drivers and control circuitry.

While liquid crystals provide large analog phase shifts with low static power consumption, their millisecond-scale dynamics limit applicability in high-speed beam steering and communication systems. Faster electrical tuning can be achieved by embedding lumped semiconductor devices, most prominently varactors and PIN diodes, directly within each meta-atom.

#### 4.1.2. Varactor and PIN-Diode Integration

(1) Basic Principle. The principle of diode-based electrical tuning is rooted in the bias-dependent modulation of the effective reactance of a resonant meta-atom. When a reverse-bias voltage *V* is applied across a varactor diode, the depletion region widens, thereby reducing the junction capacitance $C_{j}\left(V\right)$ and blue-shifting the resonance frequency of the meta-atom. This capacitive tuning provides a continuous control mechanism, in contrast to PIN diodes, which operate as binary switches by toggling between conductive (ON) and insulating (OFF) states to discretely alter the effective surface impedance. Together, these devices enable continuous or discrete electrical reconfigurability of metasurfaces [33,129,130].

(2) Phase-Control Method. From a circuit perspective, the tunability of varactor-loaded metasurfaces can be modeled by the resonance condition of an LC circuit:

(10) $$\omega_{0}\left(V\right)=\frac{1}{\sqrt{L\left(C_{0}+C_{j}\left(V\right)\right)}},$$

where *L* is the effective inductance of the resonant element, $C_{0}$ is the structural capacitance, and $C_{j}\left(V\right)$ is the bias-dependent junction capacitance of the varactor. The phase response of such a resonance follows a Lorentzian form,

(11) $$\phi \left(\omega \right)=\arctan\;\left(\frac{\gamma \omega }{\omega_{0}^{2}\left(V\right)-\omega^{2}}\right),$$

where γ is the damping rate. Reducing $C_{j}\left(V\right)$ increases $\omega_{0}\left(V\right)$, enabling voltage-dependent phase shifts that directly translate to beam steering or waveform control.

This model has been widely used to describe bias-controlled frequency shifts in varactor-based metasurfaces, as demonstrated in Fano-resonant asymmetric split-ring resonators [113], tunable radar absorbers [131], and reconfigurable dipole-type dogbone elements [132]. In particular, the practical ASRR unit-cell layout and bias routing are shown in Figure 4c, while the corresponding transmission-phase and group-delay spectra, exhibiting steep, bias exploitable dispersive slopes around the resonance, are plotted in Figure 4d. In each case, the bias-driven reduction in $C_{j}\left(V\right)$ produces a blue shift in the resonance, consistent with the inverse square-root dependence in (10).

(3) Limitations. More refined descriptions account for varactor parasitics. The small-signal equivalent circuit of a packaged varactor incorporates series resistance $R_{s}$, package inductance $L_{p}$, and parasitic capacitance $C_{p}$, which significantly influence the quality factor and impedance bandwidth. Howard [132] captured these effects through an equivalent-circuit model combined with SPICE-based diode modeling, enabling accurate prediction of scattering parameters without exhaustive full-wave simulations. Similarly, López [131] emphasized the role of bias-dependent resistance in addition to capacitance, showing that absorption bandwidth can be optimized by jointly tuning both. A practical trade-off is that increased bias depth improves phase tunability but also increases resistive loss, limiting efficiency at large tuning ranges.

(4) Practical Implementations and Application Scenarios. In contrast, PIN diodes simplify the response to two quantized impedance states,

(12) $$Z_{s}=\left\{\begin{array}{cc} R_{\mathrm{on}}, & \mathrm{ON}\;\mathrm{state}\;\left(\mathrm{conductive}\right)\\ R_{\mathrm{off}}\|\left(1/j\omega C_{j}\right), & \mathrm{OFF}\;\mathrm{state}\;\left(\mathrm{insulating}\right), \end{array}\right.$$

which is particularly suited for digital coding metasurfaces and programmable holography [129,133]. PIN-diode metasurfaces have demonstrated real-time beam steering and RIS in the microwave regime [34,134], where fast ON and OFF currents enable sub-microsecond switching.

Representative ranges for achievable phase shift, tuning speed, and insertion loss reported for diode-based electrically tunable metasurfaces are summarized in Table 4.

Although varactor and PIN-diode architectures enable sub-microsecond reconfiguration, their performance at optical frequencies is constrained by parasitic reactances, resistive loss, and fabrication complexity. To achieve ultrafast phase modulation in the optical and near-infrared regime, metasurfaces increasingly leverage intrinsic material nonlinearities, most notably the Pockels effect in electro-optic crystals. Building on the limitations of lumped electrical tuning at optical frequencies, electro-optic metasurfaces exploit the Pockels effect to achieve ultrafast, bias-controlled phase modulation without discrete circuit elements.

#### 4.1.3. Electro-Optic Modulation

Electro-optic metasurfaces implement this concept by exploiting the Pockels effect in non-centrosymmetric materials to achieve ultrafast, electrically controlled phase modulation at optical and near-infrared frequencies.

**(1) Basic Principle.** Electro-optic modulation in metasurfaces is based on the Pockels effect, where the refractive index of a non-centrosymmetric medium (such as LiNbO_3_ or BaTiO_3_) varies linearly with the strength of an applied electric field [65,135]. The principle originates from the field-induced modification of the dielectric permittivity tensor, expressed as

(13) $$\Delta \;{\left(\frac{1}{n^{2}}\right)}_{i}=\sum_{j}r_{ij}E_{j},$$

where *n* is the refractive index, $r_{ij}$ is the electro-optic coefficient tensor, and $E_{j}$ is the applied electric field component along the *j*-axis. This relation indicates that the refractive index change Δn is proportional to $E_{j}$, enabling continuous and ultrafast modulation of optical phase.

(2) Phase-Control Method. For a wave propagating through an EO medium of thickness *d*, the induced phase shift can be written as

(14) $$\Delta \phi =\frac{2\pi }{\lambda }\;n^{3}r_{ij}E_{j}\;d,$$

where λ is the free-space wavelength. This expression shows that metasurface phase tuning scales with both the intrinsic electro-optic coefficient and the applied electric field, enabling analog and high-resolution phase control.

When integrated with metasurfaces, resonant elements such as nanoantennas or subwavelength gratings are either fabricated directly on electro-optic substrates or embedded within thin electro-optic films. The local refractive index modulation alters the effective optical path length of each resonator, thereby tuning its resonance frequency and phase response. Unlike varactor- or PIN-diode-based approaches, electro-optic modulation does not rely on lumped circuit elements but instead leverages intrinsic material nonlinearities, enabling sub-nanosecond response times that are primarily limited by the RC constants of the electrodes [136,137,138,139].

Representative ranges for achievable phase modulation depth, tuning speed, and insertion loss reported for electro-optic metasurfaces are summarized in Table 4.

(3) Practical Implementations. This mechanism has been exploited in various high-speed optical and near-infrared metasurfaces. Wang et al. [36] demonstrated gigahertz-speed electro-optic modulation in LiNbO_3_-integrated metasurfaces. Karvounis et al. [140] reported electrically tunable optical metasurfaces using BaTiO_3_ thin films, while Yu et al. [141] achieved reconfigurable metasurface holography through electro-optic phase tuning. These demonstrations highlight that electro-optic modulation is well suited for high-speed beam steering, intensity modulation, and programmable holography, where switching faster than microsecond time scales is required.

As a representative implementation, Figure 4e,f shows the practical device layout and the measured phase–voltage response reported by Leng et al. [114]. Using a lithium-niobate-on-insulator metasurface with a transparent top electrode and reflective backplane, nearly 2π continuous phase modulation is achieved at telecom wavelengths while maintaining high reflectance, illustrating material-intrinsic, lumped-element-free electro-optic control.

(4) Limitations. Electro-optic modulation requires sufficiently high electric fields to induce appreciable refractive-index change, which often necessitates nanoscale electrode gaps or elevated drive voltages [36,140]. Electrode absorption and RC delays further reduce modulation efficiency at optical frequencies [136,137], and fabrication of tightly spaced electrodes on electro-optic substrates increases process complexity [138]. Despite these constraints, electro-optic platforms remain the fastest tunable metasurfaces available, with demonstrated sub-nanosecond switching [36,139], making them uniquely suited for real-time dynamic wavefront control.

Electro-optic platforms therefore offer unmatched modulation speed but require carefully engineered electrodes and relatively large bias fields. An alternative route to compact, broadband, and electrically agile tunability exploits atomically thin materials, most prominently graphene, whose conductivity and optical phase response can be reconfigured through Fermi-level gating.

#### 4.1.4. Graphene and 2D Material Gating

Complementary to electro-optic modulation, graphene and other two-dimensional materials enable electrically reconfigurable metasurfaces through carrier-density control in atomically thin platforms. Graphene and other two-dimensional (2D) materials offer exceptional electrical tunability due to their gate-dependent Fermi level, which directly modulates their optical conductivity. Unlike bulk materials, graphene is characterized by a surface conductivity σ(ω) rather than a volumetric permittivity. This conductivity arises from both intraband and interband electronic transitions and can be dynamically tuned through electrostatic gating.

The optical conductivity of graphene is commonly described by Kubo formalism [142,143,144]:

(15) $$\sigma \left(\omega ,\mu_{c},\Gamma ,T\right)=\sigma_{\mathrm{intra}}\left(\omega ,\mu_{c},\Gamma ,T\right)+\sigma_{\mathrm{inter}}\left(\omega ,\mu_{c},\Gamma ,T\right),$$

where $\mu_{c}$ is the chemical potential (Fermi level), Γ is the scattering rate, *T* is the temperature, and ω is the angular frequency. The intraband term dominates in the terahertz regime and is given by

(16) $$\sigma_{\mathrm{intra}}\left(\omega \right)=\frac{2ie^{2}k_{B}T}{\pi \hbar^{2}\left(\omega +i\Gamma \right)}\ln\;\left[2\cosh\;\left(\frac{\mu_{c}}{2k_{B}T}\right)\right],$$

while the interband contribution becomes significant at near-infrared and optical frequencies, with an absorption edge around $2\mu_{c}$. By adjusting $\mu_{c}$ through gate bias, the balance between these two mechanisms shifts, enabling dynamic control over absorption, reflection, and phase modulation.

This tunable surface conductivity allows graphene metasurfaces to achieve reconfigurable electromagnetic responses with subwavelength resolution. Gate-controlled modulation has been demonstrated across terahertz to mid-infrared frequencies, enabling functionalities such as beam steering, focusing, and amplitude and phase control [37,145,146]. As a concrete demonstration, Figure 4i,j pairs the gate-tunable graphene gold metasurface layout with the measured reflection phase versus Fermi level, showing more than $230^{\circ }$ mid-infrared phase modulation under electrostatic bias [115].

Representative ranges for achievable phase modulation depth, tuning speed, and insertion loss reported for graphene and other two-dimensional-material-based metasurfaces are summarized in Table 4.

Beyond graphene, transition metal dichalcogenides (TMDCs) such as MoS_2_ and WS_2_ provide complementary functionalities. Their strong excitonic resonances in the visible and near-infrared can be electrically tuned through gating, opening opportunities for hybrid two-dimensional metasurfaces that combine broadband graphene conductivity control with excitonic phase and amplitude modulation [147,148,149]. Such hybrid approaches leverage the ultrathin nature of two-dimensional materials, broadband tunability, and CMOS compatibility, making carrier-density gating a powerful mechanism for dynamic metasurface control.

While two-dimensional materials offer fast electrical tuning and large modulation depth, their optical loss and challenges in achieving uniform large-area integration can limit overall device efficiency. These considerations naturally motivate alternative dynamic platforms based on geometric deformation rather than carrier-density control. We therefore next examine mechanical tuning, where strain or bending modifies the resonant pathways of a metasurface to achieve phase reconfiguration.

### 4.2. Phase Manipulation via Mechanical Deformation

Mechanical deformation modulates optical phase by altering the optical path difference experienced by light,

(17) $$\phi =\frac{2\pi }{\lambda }\;\mathrm{OPD},$$

a fundamental relation in wave optics that underpins deformation-driven phase control. In mechanically reconfigurable metasurfaces, the optical path difference varies through two coupled strain-driven channels: (i) geometric transformation, including changes in lattice spacing, thickness, or orientation, which modify the physical path length, and (ii) effective-index modulation, arising from strain-induced changes in the local dielectric environment, modal confinement, and inter-element coupling.

For small deformations (|ϵ|≪1), the strain-induced phase change can be expressed in compact form as

(18) $$\Delta \phi =\frac{2\pi }{\lambda }\;\Delta \mathrm{OPD}=\frac{2\pi }{\lambda }\;\Delta \;\left(n_{\mathrm{eff}}\;h\right),$$

where $n_{\mathrm{eff}}$ is the mode-averaged effective refractive index and *h* is the effective optical thickness of the metasurface or resonant layer. This formulation follows standard wave-optics and guided-wave treatments of phase accumulation and strain-induced optical modulation [150,151,152]. It highlights that phase modulation originates from both changes in physical path length and strain-induced variations in effective refractive index, consistent with photoelastic and optomechanical descriptions of deformable optical systems [153,154].

Early frameworks organized mechanical reconfiguration strategies such as substrate stretching and microelectromechanical actuation, establishing continuous and reversible tuning of metasurface resonances for beam shaping and holography [155,156]. Subsequent demonstrations on deformable polymeric substrates confirmed that controlled strain can tune metasurface resonances and phases, enabling reconfigurable metalenses, metaholograms, and structural-color devices [157,158]. To mitigate absorption losses inherent to plasmonic platforms, recent efforts have shifted toward low-loss all-dielectric metasurfaces, where strain simultaneously modifies lattice geometry and inter-element coupling to yield efficient phase control.

**Figure 5:**
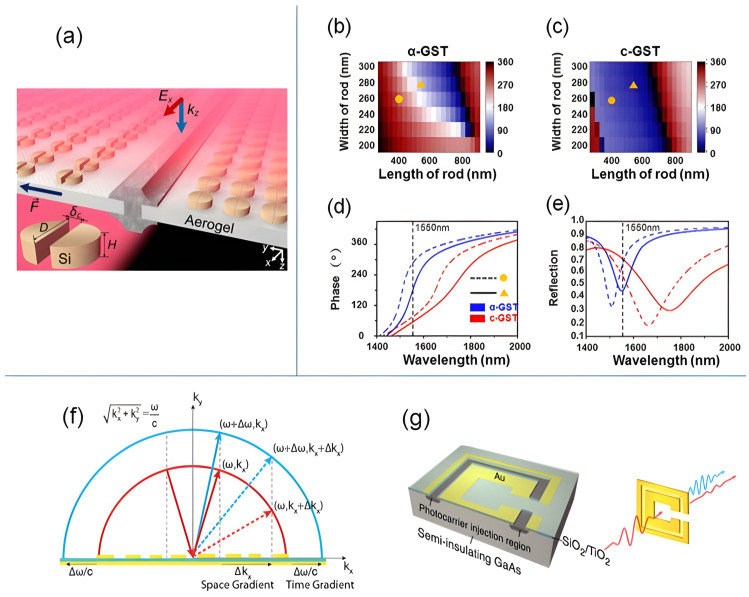
Overview of additional dynamic phase-control mechanisms discussed in subsequent sections. (**a**) Mechanical deformation: stretchable all-dielectric metasurface where applied strain modifies lattice spacing and coupling, shifting the resonance and scattered phase (adapted from [39]). (**b**–**e**) Phase-change metasurfaces: geometry–phase maps and spectra for GST nanorods in amorphous and crystalline states, illustrating nonvolatile phase reprogramming near 1550 nm (adapted from [38]). (**f**) Space–time modulation principle: combined spatial and temporal phase gradients produce momentum and frequency shifts, enabling nonreciprocal and frequency-agile control (adapted from [41]). (**g**) Device-level temporal modulation: optically pumped THz metasurface where transient photocarriers induce time-varying phase for frequency conversion (adopted from [51]).

As a representative example, Figure 5a, adapted from Prokhorov et al. [39], shows a stretchable all-dielectric metasurface in which applied strain changes lattice spacing and coupling strength, shifting the resonance and thereby modifying the accumulated optical phase. In resonant metasurfaces, this phase modulation is primarily governed by resonance detuning, with the phase following the steep local dispersion rather than a purely propagation-based accumulation. Accordingly, even when experimental reports emphasize spectral shifts, the associated phase response can be inferred from the resonance dispersion.

Scalability has also been demonstrated in the terahertz regime, where a PDMS-based metasurface absorber fabricated via screen printing exhibited a reversible resonance redshift of up to 1.51 THz under 25% applied strain [159].

In summary, mechanical deformation provides a passive, continuous, and reversible route to phase control by jointly tailoring physical path length and effective refractive index, frequently mediated through resonance detuning. While actuation speed, strain uniformity, and mechanical fatigue remain practical constraints, this approach is particularly attractive for flexible, broadband, large-area, and biocompatible photonic systems.

### 4.3. Phase-Change Materials

Phase-change materials such as Ge_2_Sb_2_Te_5_ enable non-volatile and reversible transitions between amorphous and crystalline phases, providing large refractive-index contrast for reconfigurable flat optics. The index shift, typically Δn≈1 to 2 in the near infrared, induces a phase change

(19) $$\Delta \phi =\frac{2\pi }{\lambda }\;\Delta n\;t,$$

where *t* is the phase-change-material thickness. This large intrinsic contrast enables compact phase modulation compared with electro-optic or carrier-density-based platforms, although optical absorption and thermally driven switching remain key considerations [160,161].

Early demonstrations established binary switching between amorphous and crystalline states, enabling rewritable metasurface phase profiles [38]. Figure 5b–e illustrate this principle, where phase-change-induced refractive-index variation reshapes the reflection phase near 1550 nm. To reduce optical loss, lower-loss chalcogenides were subsequently developed. Sb_2_S_3_ metasurfaces achieved nearly 2π phase modulation in the visible rangewith high transmission [125], while Sb_2_Se_3_ enabled multilevel phase operation with tuning ranges exceeding π and reflectance above 0.5 across a bandwith of tens of nanometers [162].

To address limitations associated with thermal switching speed and endurance, recent work has explored all-optical and cavity-assisted phase-change platforms. Thin-film GeTe and GeSe stacks reduce optical loss and enable mixed amplitude and phase or phase-only modulation at lower switching energies [163]. These strategies aim to improve switching efficiency, scalability, and spectral selectivity while preserving non-volatility.

**Table 4:** Mechanism-level summary of representative quantitative performance ranges for active phase-control approaches. To address concerns regarding broad performance ranges and metric ambiguity, this table provides a conceptual comparison across mechanisms, while a detailed band- and application-resolved per-paper quantitative summary with explicitly defined efficiency metrics and bandwidth values is provided in Supplementary Table S2, where all figures are reported exactly as stated in the cited works. The corresponding references for these data are included in the Supplementary Materials.

Mechanism	Phase Range	Insertion Loss	Notes (typical Band/Remark)	References
Varactor/PIN diodes	∼1-bit to near 2π	∼0.2–3 dB (state dependent)	RF/microwave; bandwidth often resonance-limited per state; widely used in RIS, beam steering, and programmable reflectarrays	[134,164,165]
Liquid crystal (LC)	∼$180^{\circ }$ to 2π	∼1–5 dB	VIS–NIR; large analog tuning range; dynamics limited by LC reorientation	[89,116,125]
Electro-optic (Pockels)	up to ∼π (typ.), up to 2π (res.-enhanced)	∼3–6 dB	NIR/telecom; resonance-enhanced phase tuning; high-*Q* modes improve efficiency but limit bandwidth	[136,138,166]
Graphene/2D gating	up to ∼$200^{\circ }$–$240^{\circ }$	high near resonance (absorption dominated)	THz–mid-IR; electrically tunable surface conductivity; strong tunability offset by resonance loss	[115,167]
Phase-change materials (PCMs)	>π (multi-level) to 2π	moderate (material dependent)	NIR–THz; non-volatile multilevel programmability; suitable for static or quasi-static reconfigurable optics	[162,168,169]
Mechanical deformation	up to 2π	low (platform dependent)	Flexible and large-area platforms; performance limited by mechanical stability and uniformity	[39,170]

In summary, phase-change materials provide compact, non-volatile, and multilevel phase programmability from the visible to the infrared. Their thermally driven switching distinguishes them from electro-optic and carrier-based mechanisms; however, ongoing materials engineering and optical activation strategies continue to mitigate absorption and endurance constraints.

### 4.4. Comparative Analysis of Active Phase-Control Mechanisms

Table 4 highlights that, despite partially overlapping numerical ranges, the underlying performance trade-offs of active phase-control mechanisms are fundamentally distinct. Varactor- and PIN-diode metasurfaces provide mature and electrically addressable reconfiguration pathways with near-full 2π phase coverage, making them particularly effective for RF beam steering, programmable reflectarrays, and RIS implementations. Liquid-crystal-based metasurfaces achieve similarly large phase excursions with very low static power consumption and analog tunability, although their operation relies on molecular reorientation processes that restrict applicability in rapidly reconfigurable systems.

Electro-optic metasurfaces exploiting the Pockels effect offer intrinsically fast index modulation and are therefore well suited for high-speed optical wavefront control. However, the achievable phase range is often limited to ∼π unless resonance-enhanced architectures are employed, and practical performance is influenced by electrode integration and resonance bandwidth. Carrier-density tuning in graphene and related materials extends electrical phase control into the terahertz and mid-infrared regimes, providing substantial tunability at the expense of increased absorption near resonant conditions.

Phase-change materials uniquely combine large phase contrast with non-volatility, enabling multilevel and reprogrammable optical apertures that retain their state without continuous biasing. Finally, mechanically reconfigurable metasurfaces enable low-loss and large-area phase modulation through geometric deformation, although their actuation mechanism inherently favors quasi-static or flexible photonic applications.

Taken together, the quantitative ranges summarized in Table 4 underscore that no single active mechanism is universally optimal. Instead, practical metasurface design requires balancing phase range, loss characteristics, volatility, integration complexity, and application bandwidth in accordance with the targeted frequency band and system-level requirements.

### 4.5. Space–Time Phase Modulation

#### 4.5.1. Conceptual Distinction from Quasi-Static Active Tuning

Unlike the active tuning mechanisms discussed in the previous section—where the metasurface is reconfigured between discrete and stable phase states—space–time modulation (STM) operates in a fundamentally dynamic regime. In electrically, mechanically, or phase-change-based active tuning, an external stimulus modifies the local electromagnetic response until a new steady-state configuration is reached. Once programmed, the output wave maintains the same carrier frequency, and the metasurface functions as a quasi-static spatial phase transformer.

In contrast, STM introduces a deliberate temporal variation in the surface impedance or phase response, such that the phase profile becomes explicitly dependent on both position and time, ϕ(x,t). The presence of a nonzero temporal gradient ∂ϕ/∂t≠0 enables energy exchange between different frequency components, generating harmonic sidebands and allowing frequency translation. Consequently, STM metasurfaces dynamically redistribute energy in the frequency–momentum domain, enabling magnetless nonreciprocity, frequency mixing, and duplex communication—functionalities fundamentally inaccessible to quasi-static reconfiguration.

For clarity, the essential differences are summarized in Table 5.

Space–time modulation extends a metasurface’s static phase profile by introducing a designed temporal dependence, enabling joint control of tangential momentum and optical frequency. As shown in Figure 5f, adapted from [41], a spatial phase gradient imparts in-plane momentum while a temporal phase gradient induces frequency translation:

(20) $$\Delta k_{x}=\frac{\partial \phi }{\partial x},\;\Delta \omega =-\;\frac{\partial \phi }{\partial t}.$$

Accordingly, an imposed phase profile ϕ(x,t) maps an incident state $\left(\omega ,k_{x}\right)$ to (ω+Δω,$k_{x}+\Delta k_{x})$, forming the basis for momentum–frequency coupling in STM platforms [40,41,171].

#### 4.5.2. Reciprocity and Traveling-Wave Modulation

In linear time-invariant systems, electromagnetic reciprocity follows from the Lorentz reciprocity theorem and imposes the scattering-matrix symmetry condition

(21) $$S=S^{T}.$$

Nonreciprocity requires violation of this symmetry,

(22) $$S\neq S^{T},$$

which can only occur when time-reversal symmetry is broken. Purely temporal modulation (K=0) results in symmetric frequency conversion and therefore preserves reciprocity. In contrast, traveling-wave modulation—requiring both Ω≠0 and K≠0—produces asymmetric indirect photonic transitions that enable direction-dependent scattering [40,172].

#### 4.5.3. Harmonic Generation and Floquet Picture

A traveling-wave impedance model provides an intuitive description of STM dynamics. If the surface impedance is modulated as

(23) $$Z_{s}\left(x,t\right)=Z_{0}+\Delta Z\cos\left(\Omega t-Kx+\psi \right),$$

the scattered fields contain discrete space–time Floquet harmonics:

(24) $$\omega_{n}=\omega_{0}+n\Omega ,\;k_{x,n}=k_{x,0}+nK.$$

Temporal modulation enables frequency conversion, while the spatial component controls the directionality of each harmonic order. Selective enhancement of desired sidebands can be achieved via traveling-wave biasing, serrodyne modulation, or IQ-controlled feeding schemes [44,171,173].

**Figure 6:**
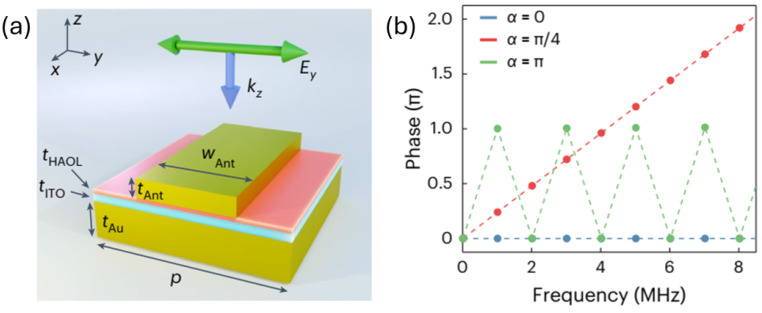
(**a**) Device stack of an electrically biased ITO-based STM metasurface, consisting of an Au back reflector, ITO active layer, HAOL gate dielectric, and Au nanoantenna. (**b**) Harmonic phase response for different temporal offsets α, demonstrating the linear relation $\phi_{n}=n\alpha$ (adapted from [175]).

A practical realization of electrically driven STM is shown in Figure 6. The metasurface employs a plasmonic MOS stack in which voltage-induced carrier accumulation in the ITO layer enables megahertz-scale temporal index modulation. Two interleaved electrode groups are driven with identical waveforms but a controlled temporal offset α, converting temporal modulation into a spatially varying harmonic phase. The generated sidebands obey the linear phase relation $\phi_{n}=n\alpha$, enabling programmable spatial gratings for each frequency component. Experimentally, clean harmonics at $\omega_{0}\pm n\Omega$ are observed with more than 20 dB suppression of unwanted orders, and approximately 94% of the 1 MHz sideband power is diffracted into targeted spatial modes.

#### 4.5.4. System-Level Implications and Practical Considerations

STM concepts further extend to digitally programmable space–time-coding (STC) metasurfaces for harmonic beam steering and frequency–space multiplexing in beyond-5G/6G systems [42,174]. Practical implementation, however, is constrained by modulation bandwidth, driver synchronization, harmonic purity, and absorption associated with rapid conductivity modulation [51].

Unlike static or quasi-static phase-control mechanisms, space–time modulation introduces an independent temporal degree of freedom that couples energy across frequencies and propagation directions. STM metasurfaces therefore extend passive wavefront engineering toward dynamically programmable platforms capable of frequency conversion, nonreciprocal transport, and spectral–spatial multiplexing.

## 5. Power Allocation and Control Frameworks for Active Metasurfaces

Having surveyed the mechanisms by which metasurfaces achieve active and reconfigurable phase control, from electrically tunable unit cells to hybrid and space–time-modulated platforms, it is equally important to examine how large programmable apertures are addressed, powered, and dynamically calibrated in practical implementations.

Electrically tunable metasurfaces employ several addressing schemes that strongly influence scalability and control complexity. Liquid-crystal-based devices commonly use a row–column passive-matrix configuration, where each pixel is selected at the intersection of a driven row and column, and the liquid-crystal layer provides extremely low static power consumption [176]. Digital coding metasurfaces often adopt column-level or block-level addressing, in which PIN-diode states are updated by a field-programmable gate array to synthesize programmable far-field patterns or enable direct digital message transmission [177]. More modular architectures implement tile-level control, where each tile or block of meta-atoms operates as an independently programmable unit accessed through a distributed controller, enabling scalable deployment across large apertures. At higher integration levels, application-specific integrated circuit-enabled metasurfaces provide per-pixel or per-cell analog programmability using tunable resistive–capacitive loads and on-chip digital-to-analog converters, allowing fine impedance control and predictable power allocation across extensive apertures [178,179,180].

Feedback and calibration strategies likewise vary across platforms. Sensor-integrated metasurfaces incorporate local photodetectors or sensing elements to autonomously adjust coding states in response to external stimuli, as demonstrated in ultraviolet-controlled metasurfaces where each supercell reports its optical intensity to a controller for automatic state selection [181]. Digital coding metasurfaces also support closed-loop optimization by iteratively updating control patterns based on received-signal metrics, as in adaptive wireless power-transfer systems where beamforming codewords are refined using over-the-air feedback [182]. More advanced implementations employ machine-learning-based controllers, in which neural-network-derived decision models or learned measurement modes directly determine the metasurface configuration in real time. Representative examples include machine-learning-guided programmable imagers [183] and deep-learning-driven intelligent metasurfaces capable of autonomously updating their scattering response in dynamic environments [184].

## 6. Practical Application Challenges Across Metasurface Mechanisms

While the preceding sections systematically analyzed the fundamental phase-control mechanisms, from resonance-based and PB phase approaches to hybrid and active modulation strategies, their practical realization extends beyond physical design principles. Translating these mechanisms into functional metasurface platforms requires addressing fabrication scalability, material compatibility, system-level integration, power efficiency, response speed, and operational reliability. The following subsections examine these implementation-driven constraints and highlight how they shape the technological maturity and application readiness of each mechanism.

### 6.1. Scalability and Fabrication

Subwavelength plasmonic resonators at visible frequencies require high-resolution nanolithography and tight dimensional control, posing scalability and uniformity challenges for large-area fabrication [5,185,186]. Multi-layer or cascaded metalens designs improve aberration correction and efficiency but significantly increase fabrication complexity [10,95]. PB-phase metasurfaces require precise rotational alignment of anisotropic meta-atoms, where fabrication tolerances directly impact phase accuracy [187].

Near-IR Huygens’ metasurfaces require precise nanodisk geometries, where fabrication imperfections reduce transmission efficiency [26,188]. At Ku-band, varactor size becomes non-negligible, introducing additional losses and layout constraints unless carefully integrated [164]. LC-based devices face challenges in pixel pitch reduction and crosstalk suppression [125].

Lithium niobate offers high electro-optic coefficients but thin-film processing and etching remain technologically demanding [36]. ITO-based ENZ metasurfaces enable efficient electrical modulation but are constrained by gate leakage and dielectric breakdown considerations [189]. Hybrid ENZ metasurfaces require precise spectral alignment between plasmonic resonances and ENZ conditions [190]. Graphene-gold devices are limited by substrate losses and dielectric breakdown in gating layers [115].

GST-based PCM rods require precise energy delivery for selective crystallization [38]. Low-loss Sb_2_Se_3_ nanostructures demand uniform lithography control to ensure reproducible optical states [191]. GeTe thin films must be carefully optimized in thickness to balance amplitude modulation and optical loss [163]. CMOS compatibility varies across platforms: graphene and ITO integrate more readily, while LN processing remains comparatively complex.

### 6.2. System Integration

Packaging and electromagnetic coupling are critical challenges. Embedding antennas in dielectric environments shifts resonance wavelengths depending on local refractive index [14]. LC devices require encapsulation layers to maintain optical efficiency and mechanical stability [122]. Ku-band varactor reflectors demand careful bias routing to minimize parasitic losses [164].

Graphene and ITO devices rely on transparent conductive electrodes for electrical gating without obstructing optical paths [115,189]. ENZ-hybrid metasurfaces integrate plasmonic SRRs with conductive oxide films to enable nonlinear THz generation [190]. Pixel-level addressing for LC SLMs and PCM arrays requires dense interconnect architectures, complicating large-area scaling. PCM devices further require encapsulation layers to prevent oxidation and structural degradation during repeated switching [163].

### 6.3. Power Consumption

PIN-diode metasurfaces reduce insertion loss with higher bias current but increase static power consumption [134]. LC devices typically require continuous bias to maintain molecular orientation, increasing average energy usage [122].

In contrast, non-volatile PCMs such as GST and Sb_2_Se_3_ consume power only during switching events, improving energy efficiency [38,191]. Graphene and ITO devices operate via capacitive gating mechanisms requiring comparatively low modulation energy [115,189]. GeTe amplitude-dominant modulators enable efficient operation with minimal steady-state power draw [163].

### 6.4. Response Speed

Liquid crystals are fundamentally limited by molecular reorientation times in the millisecond regime, restricting high-speed modulation [122,125]. Varactor-based RF metasurfaces enable continuous tuning with faster response than PIN diodes but typically exhibit limited phase range [164].

Electro-optic materials such as lithium niobate enable sub-nanosecond Pockels-based modulation [36]. ITO ENZ modulators operate in the hundreds of kHz regime depending on device capacitance [189]. Graphene-based modulators demonstrate potentially GHz-scale operation under optimized gating conditions [115]. PCM switching speeds range from nanoseconds (optical excitation) to microseconds depending on heating architecture [38,191]. ENZ-hybrid nonlinear metasurfaces enable ultrafast THz generation via femtosecond excitation [190].

### 6.5. Reliability & Stability

Mechanical tuning approaches may suffer from fatigue and structural wear over extended cycling. LC materials degrade under prolonged bias or UV exposure [122]. Oxide-based gating devices face dielectric breakdown and charge trapping issues [189]. Graphene devices may exhibit hysteresis due to interface states [115].

PCM endurance is finite but materials such as Sb_2_Se_3_ and GeTe demonstrate reliable multi-cycle switching performance when properly encapsulated [163,191]. Protective dielectric capping layers mitigate oxidation and mechanical degradation during phase transitions.

### 6.6. Comparative Outlook

Trade-offs remain mechanism-dependent. Liquid crystals are mature and versatile but limited in speed. RF diodes and varactors are efficient at microwave frequencies but unsuitable for optical regimes. Electro-optic crystals offer ultrafast response but require advanced fabrication infrastructure. Graphene and ITO enable fast electrical tuning but face modulation-depth and breakdown constraints. Phase-change materials uniquely combine non-volatility and multi-level control, though uniformity and endurance remain design challenges [38,163,191]. Each platform balances scalability, speed, efficiency, and reliability differently, making them complementary rather than competing solutions.

## 7. Conclusions and Outlook

Metasurfaces have transformed electromagnetic control from bulky refractive optics into ultrathin platforms capable of sculpting phase, amplitude, and polarization with subwavelength granularity [1,2,3]. Static phase-control mechanisms, including resonant, PB, and propagation-based approaches, established the foundations of flat optics, each offering distinct trade-offs among bandwidth, dispersion, efficiency, and polarization selectivity [16,18,85]. Subsequent advances in Huygens-type and hybrid metasurfaces addressed efficiency and bandwidth limitations of early plasmonic designs by co-engineering electric and magnetic responses and exploiting multipolar and cavity-assisted effects [26,30,70,192].

More recently, the field has progressed beyond static wavefront engineering toward dynamically reconfigurable and programmable electromagnetic control. Tunable material platforms, including liquid crystals, electro-optic oxides, ENZ conductors, graphene, and phase-change chalcogenides, enable post-fabrication phase reconfiguration and transform metasurfaces into adaptive optical components [112,162]. Space–time modulation further introduces an explicit temporal degree of freedom, coupling spatial and temporal phase gradients to realize nonreciprocity, frequency agility, and waveform synthesis beyond the capabilities of static or quasi-static platforms [40,41,43,45,171,193]. Together, these developments mark a conceptual transition from passive phase plates to actively reconfigurable and time-varying electromagnetic processors.

Looking ahead, several research directions are expected to shape the continued evolution of metasurfaces. First, broadband and dispersion-aware design strategies based on inverse-designed, hybrid, and multipolar meta-atoms are likely to enable practical achromatic beamforming and metalenses without sacrificing efficiency [70,97,98,192]. Second, continued progress in low-loss and reconfigurable materials, including refined ENZ oxides, low-loss chalcogenides, and emerging two-dimensional semiconductors, promises larger and more reversible refractive-index modulation with reduced absorption [37,112,162,194]. Third, electro-optic and carrier-driven platforms are expected to advance toward sub-microsecond modulation speeds with reduced drive voltages, supported by optimized electrode design, transparent conductors, and improved RC architectures [114,138,195].

At the system level, programmable space–time metasurfaces and digital coding architectures are expected to play an increasingly central role in nonreciprocal beam control, frequency-multiplexed communication, adaptive sensing, and emerging 6G-class wireless and terahertz systems [41,45,171,193]. Rather than relying on isolated performance metrics, future progress will hinge on holistic co-design of meta-atoms, materials, control electronics, and feedback or learning-based algorithms, emphasizing stability, scalability, and system-level functionality [196,197].

In summary, metasurfaces are converging toward intelligent electromagnetic platforms that unify materials innovation, high-quality-factor meta-atom engineering, scalable fabrication, high-speed and low-power electronics, and data-driven control. This convergence positions metasurfaces not only as compact optical components, but as adaptive technologies capable of shaping both electromagnetic waves and information itself [3,4].

## Figures and Tables

**Table 1:** Comparison of representative metasurface phase-control review articles with this work.

Feature/Scope	[52]	[47]	[48]	[49]	[50]	This Work
Static phase mechanisms (resonant, PB, propagation)	✓	✓	✓	✓	✓	✓
Hybrid phase mechanisms	×	Partial	×	Partial	Partial	✓
Active tuning mechanisms	Limited	✓	✓	✓	✓	✓
Space–time modulation	×	×	×	×	Partial	✓
Mechanism-centric organization	×	Partial	Partial	Partial	Partial	✓
Unified theoretical perspective	×	×	×	×	Partial	✓
Recent literature coverage (2020–2025)	×	Partial	Partial	Partial	✓	✓
Comparative synthesis and tables	Limited	Limited	Limited	Limited	Partial	✓

**Table 5:** Key distinction between quasi-static active tuning and space–time modulation.

Characteristic	Active Tuning	STM
Temporal gradient ∂ϕ/∂t	No	Yes
Carrier frequency conserved	Yes	No
Floquet harmonics generated	No	Yes
Reciprocity breaking	No	Yes *
Operational regime	Quasi-static	Time-varying

* Requires traveling-wave modulation (Ω≠0, K≠0).

## Data Availability

All the data generated or analyzed during this study are included in this published article.

## Supplementary Materials

The following supporting information can be downloaded at https://www.mdpi.com/article/10.3390/s26061781/s1.

## Nomenclature

Acronyms used in this manuscript.			
**Acronym**	**Definition**	**Acronym**	**Definition**
2D	Two-dimensional	LC	Liquid crystal
3-dB	Three-decibel (half-power) bandwidth	LN	Lithium niobate (LiNbO_3_)
5G	Fifth-generation wireless communication	LNOI	Lithium-niobate-on-insulator
6G	Sixth-generation wireless communication	MEMS	Micro-electro-mechanical systems
AI	Artificial intelligence	MOS	Metal–oxide–semiconductor
AR	Aspect ratio	MW	Microwave
ASRR	Asymmetric split-ring resonator	NA	Numerical aperture
BaTiO_3_	Barium titanate	NIR	Near-infrared
BW	Bandwidth	NV	Non-volatile
CMOS	Complementary metal–oxide–semiconductor	OAM	Orbital angular momentum
CP	Circularly polarized	OPD	Optical path difference
DC	Direct current (bias)	PB	Pancharatnam–Berry (geometric) phase
DBR	Distributed Bragg reflector	PCM	Phase-change material
d.o.f.	Degrees of freedom	PCMs	Phase-change materials
EM	Electromagnetic	PDMS	Polydimethylsiloxane
ENZ	Epsilon-near-zero	PIN	Positive–intrinsic–negative (diode)
EO	Electro-optic	RF	Radio frequency
FOM	Figure of merit	RCS	Radar cross section
GMR	Guided-mode resonance	RIS	Reconfigurable intelligent surface
GSL	Generalized Snell’s law	SLM	Spatial light modulator
GST	Ge_2_Sb_2_Te_5_	SRR	Split-ring resonator
HAOL	Al_2_O_3_/HfO_2_ nanolaminate	ST	Space–time
ITO	Indium tin oxide	STM	Space–time-modulated
TE	Transverse electric polarization	TM	Transverse magnetic polarization
THz	Terahertz	TMDC(s)	Transition metal dichalcogenide(s)
VIS	Visible spectrum		
Symbols used in this manuscript.			
**Symbol**	**Description**	**Unit**	**Context/Section**
λ	Free-space wavelength	m	Phase accumulation, dispersion analysis
*f*	Operating frequency	Hz	Microwave to optical metasurfaces
$k_{0}$	Free-space wavenumber (2π/λ)	rad/m	Generalized Snell’s law
ϕ	Phase shift imparted by metasurface	rad	All phase-control mechanisms
Δϕ	Phase gradient along surface	rad/m	Beam steering and refraction
$\theta_{i}$	Angle of incidence	deg/rad	Generalized Snell’s law
$\theta_{t}$	Angle of transmission/reflection	deg/rad	Wavefront manipulation
$n_{\mathrm{eff}}$	Effective refractive index	–	Propagation-phase metasurfaces
*h*	Meta-atom height/thickness	m	Propagation and mechanical tuning
Ω	Rabi splitting energy	eV	Strong light–matter coupling (examples)
$\epsilon_{r}$	Relative permittivity	–	Resonant and dielectric metasurfaces
σ	Surface conductivity	S	Graphene/ENZ tuning
*Q*	Quality factor of resonance	–	Resonant and hybrid mechanisms
*E*, *H*	Electric and magnetic fields	V/m, A/m	Electromagnetic formulation
$\alpha_{e}$	Electric polarizability	m^3^	Dipole scattering model
$\alpha_{m}$	Magnetic polarizability	m^3^	Huygens metasurfaces
$Z_{\mathrm{ms}}$	Magnetic surface impedance	Ω	Huygens condition
$Y_{\mathrm{es}}$	Electric surface admittance	S	Impedance matching
θ	Rotation angle of meta-atom	deg/rad	PB phase control
±2θ	PB phase shift	rad	Spin-dependent phase encoding
*V*	Applied bias voltage	V	Electrically tunable metasurfaces
*I*	Drive current	A	PIN-diode/varactor tuning
τ	Modulation time constant	s	Tuning speed comparison
