# Supplementary material for: Phase Control Mechanisms in Metasurfaces: From Static Approaches to Active and Space–Time Modulation
Source: Sensors (Basel). 2026 Mar 11;26(6):1781. doi: 10.3390/s26061781 (PMC13030159; doi:10.3390/s26061781)
Supplement: Supplementary file 1 [file sensors-26-01781-s001.zip › sensors-4161595-supplementary.pdf]

TABLE S_1: Band- and application-resolved quantitative summary for representative static phase-control mechanisms. Efficiency types: R = reflection; T = transmission; FE = focusing efficiency; AE = aperture efficiency; n.r. = not explicitly reported.

Band	Application	Mechanism	Phase Range	Efficiency	3-dB BW	Reference
**Microwave band**						
Microwave	GRIN lens / focusing	Propagation (GRIN)	Full 0–2π	$>$ 50% (AE at center frequency)	65% (gain)	[1]
Microwave	GRIN lens / focusing	Propagation (GRIN)	Full 0–2π	Max 67% (AE); $>$ 50% over X-band	66% (impedance BW)	[2]
Microwave	Beam steering / nonreciprocal	PB (magneto-optical)	Full 0–2π	Up to 96% (1st-order diffraction, forward)	n.r.	[3]
Microwave	High-efficiency lens	Huygens	Full 0–2π	$>$ 86% (T)	15–20%	[4]
Microwave	Full-space bifunctional (CP-decoupled R/T)	PB + Propagation	Full 0–2π	$\sim$ 83% (co-pol T, 9 GHz)	n.r.	[5]
Microwave	Independent CP reflection	PB + Propagation	Full 0–2π	$>$ 90% (co-pol R)	$>$ 40% (fractional BW)	[6]
**Optical band (Visible)**						
Visible	Anomalous reflection	Resonant (plasmonic)	Up to $\sim 2\pi$	$\sim$ 85% (R)	$\sim$ 85%	[7]
Visible	Metalens	Propagation (dielectric)	Full 0–2π	Up to $\sim$ 90% (T)	n.r.	[8]
Visible	Metalens	Propagation (dielectric)	Full 0–2π	$>$ 95% (T)	n.r.	[9]
Visible	PB metalens	PB (dielectric)	Full 0–2π	66–86% (FE)	n.r.	[10]
Visible	PB wavefront shaping	PB (plasmonic)	Full 0–2π	$\sim$ 13–23% (T)	n.r.	[11]
Visible	Achromatic metalens	PB + Resonance	Full 0–2π	$\sim$ 40% (T)	49%	[12]
**Optical band (NIR / Telecom)**						
NIR	Phase-gradient control	Resonant (plasmonic)	$\approx$ 0–2π	$\sim$ 40–50% (R)	few %	[13]
NIR	Analog optical computing	Resonant (GSP)	0–2π	Up to $\sim$ 80% (R)	~20%	[14]
NIR	Metalens	Propagation (dielectric)	Full 0–2π	$\sim$ 60–80% (FE)	n.r.	[15]
Telecom	Holography	Huygens	$\sim$ 0–2π	82% (T)	40 nm	[16]
NIR	Pulse shaping	Huygens	Full 0–2π	$>$ 90% (T)	n.r.	[17]

Continued on next page

TABLE S_1 – continued from previous page

Band	Application	Mechanism	Phase Range	Efficiency	3-dB BW	Reference
NIR	Spin multiplexing	PB + Resonance	Discrete 0–2π	$\sim$ 25–30% (R)	25%	[18]
NIR	Waveguide metal-ens	Propagation + Resonant	Full 0–2π	$\sim$ 16% (FE)	n.r.	[19]

TABLE S_2: Band- and application-resolved quantitative summary for representative *active* phase-control mechanisms. Insertion loss/efficiency metrics are reported as stated in the cited works (e.g., reflection magnitude, diffraction efficiency, or insertion loss in dB). "n.r." denotes not explicitly reported.

Band	Application	Mechanism	Phase Range	Insertion Loss / Eff.	3-dB BW	Special FOMs	Ref.
			**Microwave / RF band**				
Microwave / RF	Programmable coding (R)	PIN diode switching	2-bit (4 states, $\sim$90° step)	$\sim$3 dB (R mag., all states)	$\sim$15–20% (per band)	Dual-band (C/Ku); $\sim$90° $\pm$ 15° spacing	[20]
Microwave / RF	Beamforming antenna	Varactor tuning	Continuous $\sim$150° (per element)	Max AE $\sim$11%	$\sim$4% (9.6–10 GHz)	2D steering: $\pm$50° (az.), $\pm$70° (el.)	[21]
Microwave / RF	Beam steering (R)	Varactor tuning	$\sim$330° (near 2π)	1.5 dB (unit-cell R loss)	$\sim$300 MHz (per state)	Full Ku coverage by retuning	[22]
Microwave / RF	Independent phase & amplitude (R)	Varactor + PIN	0–180° (varactor)	$\|R\| \approx$ 0.3–0.8 (amp. tuning)	$\sim$100 MHz ($\sim$2.2%)	$\Delta\|R\| < 22.2\%$; phase var. $< \pm 9°$	[23]
Microwave / RF	1-bit coding (R)	PIN diode switching	$180° \pm 20°$	$<$ 0.9 dB (ON), $<$ 0.2 dB (OFF)	$\sim$8.2%	Energy conv.: $>$ 79.4% (ON), $>$ 94.4% (OFF)	[24]
Microwave / RF	Resistive coding (R)	Graphene gating	Binary π (1-bit)	$\|R\| \sim 0.33$ (at resonance)	$\sim$5% ($\sim$500 MHz)	R_s: 2500–580 Ω/sq (tuned)	[25]
Microwave / RF	Independent phase & amplitude (R)	PIN coding + graphene gating	Binary π (PIN)	$\|R\| \sim$ 0.35–0.75 (tunable)	n.r. (functional: 5.2–5.6 GHz)	R_s: 290–1600 Ω/sq (tuned)	[26]
			**Terahertz band**				
THz	Beam steering (deformation)	Liquid-crystal elastomer (opto-thermal)	Static 0–2π; dynamic steering via bending	Low–moderate (CSRR loss)	n.r. (functional: 0.48–1.1 THz)	Max steering tuning $\sim$22° at 0.68 THz	[27]
THz	Beam steering / focusing (R)	PCM (GST) switching	Abrupt $\sim \pi$ (state switching)	High reflectance (ground-backed)	n.r.	State change: amorphous $\leftrightarrow$ crystalline	[28]

Continued on next page

TABLE S_2 – continued from previous page

Band	Application	Mechanism	Phase Range	Insertion Loss / Eff.	3-dB BW	Special FOMs	Ref.
			**Optical band (Visible)**				
Visible	Wavefront shaping / holography	LC tuning	Up to $\sim$ 180–300°	$\sim$1–3 dB	n.r.	LC-overlayer dielectric metasurface	[29]
Visible	Switchable hologram / varifocal	LC + PB (TiO_2)	Full 0–2π (pol.-dep.)	$\sim$1–3 dB	n.r.	Helicity-selective intensity switching	[30]
Visible	Beam steering / holography	LC tuning (reflective)	Continuous 0–2π	$\sim$1–3 dB	n.r.	Diffraction efficiency (reported in paper)	[31]
Visible	SLM / beam steering (T)	LC-tuned Huygens	Discrete 0–2π (multi-level)	$\sim$3–5 dB	$\sim$10–20 nm	Beam steering eff. $\sim$36% (reported)	[32]
Visible	Reprogrammable holography (R)	Electromechanical MEMS (nano-kirigami)	Continuous 0–2π	n.r. (reported as high/constant)	n.r.	Pixel-addressable; voltage-driven deformation	[33]
			**Optical band (NIR / Telecom)**				
NIR / Telecom	Wavefront modulation (resonant)	Electro-optic (Pockels, $LiNbO_3$)	Up to $\sim \pi$	$\sim$3–6 dB	n.r. (narrow)	q-BIC / QBIC resonance shift	[34]
NIR / Telecom	Wavefront shaping (resonant)	Electro-optic (Pockels, $LiNbO_3$)	Up to π	$\sim$3–6 dB	n.r. (narrow)	Static field: 2.6 kV/mm (reported)	[35]
NIR (1550 nm)	Resonant quasi-BIC EO modulation	LiNbO3 + LC (integrated)	2 (at resonance)	n.r.	narrow (high-Q)	/V 0.6 nm/V	[36]
NIR	Resonant phase-gradient (high-Q)	EO (Si-on-LiNbO3)	Resonant efficiency modulation	+1 order transmission up to $\sim$ 70%	Ultra-narrow (Q 3.1×10^4)	¿10 dB diffraction contrast	[37]
			**Mid-infrared band**				
Mid-IR	Beam steering / phase tuning	Graphene gating (plasmonic)	Phase up to 237° (R)	$R \sim$ 1.5–12% (on resonance)	n.r. (narrow)	Critical coupling; steering eff. $\sim$23% (ideal)	[38]
Mid-IR	Beam switching (discrete states)	Graphene gating (dielectric)	Discrete diffraction states (no cont. 2π)	$\sim$45–55% (diff. eff., per state)	n.r. (narrow)	Directivity $>$ 98% (two-level), $>$ 90% (three-level)	[39]
			**Phase-change materials (Optical / NIR)**				
NIR	Multi-level phase modulation (R)	PCM (Sb_2Se_3)	Multi-level $> \pi$ (4-level)	$R0.5$ (typ. 0.6–0.8)	$\sim$ 42 nm (measured)	Reported: 1463–1505 nm (3 dB window)	[40]
NIR	Resonant phase tuning (R)	PCM (VO_2)	Up to $\sim$ 250°	Reflectance mod. $\sim$ 20–30%	n.r.	Joule heating (thermally limited)	[41]

Continued on next page

TABLE S_2 – continued from previous page

**Band**	**Application**	**Mechanism**	**Phase Range**	**Insertion Loss / Eff.**	**3-dB BW**	**Special FOMs**	**Ref.**
Telecom	Reconfigurable holography / OAM	PCM (GSST)	Full 0–2π (8-level)	Moderate (reported)	n.r.	Discrete 8 states; 1400–1600 nm operation (reported)	[42]

REFERENCES

[1] E. Erfani, M. Niroo-Jazi, and S. Tatu, "A high-gain broadband gradient refractive index metasurface lens antenna," *IEEE Transactions on Antennas and Propagation*, vol. 64, no. 5, pp. 1968–1973, 2016.

[2] Q.-W. Lin and H. Wong, "A low-profile and wideband lens antenna based on high-refractive-index metasurface," *IEEE Transactions on Antennas and Propagation*, vol. 66, no. 11, pp. 5764–5772, 2018.

[3] H. Pan, M. K. Chen, D. P. Tsai, and S. Wang, "Nonreciprocal pancharatnam-berry metasurface for unidirectional wavefront manipulations," *Optics Express*, vol. 32, no. 15, pp. 25632–25643, 2024.

[4] Q. Xue, X. Huang, Y. Wang, J. Gao, and Y. Liu, "Dual-polarized metalens based on huygens' metasurface," *Journal of Applied Physics*, vol. 138, no. 9, 2025.

[5] Z. Liu, Z. Wang, T. Li, J. Gu, Y. Shi, J. Zhang, H. Sun, and J. Wang, "Transmission-reflection-integrated bifunctional metasurface by hybridizing geometric phase and propagation phase," *Electronics*, vol. 14, no. 21, p. 4250, 2025.

[6] H. Shi, G. Li, L. Wang, J. Yi, X. Chen, A. Zhang, and Z. Xu, "Nonresonant propagation phase based metasurface design for independent manipulation of dual circularly polarized waves," *Journal of Applied Physics*, vol. 132, no. 16, 2022.

[7] Z. Li, E. Palacios, S. Butun, and K. Aydin, "Visible-frequency metasurfaces for broadband anomalous reflection and high-efficiency spectrum splitting," *Nano letters*, vol. 15, no. 3, pp. 1615–1621, 2015.

[8] A. Zhan, S. Colburn, R. Trivedi, T. K. Fryett, C. M. Dodson, and A. Majumdar, "Low-contrast dielectric metasurface optics," *ACS photonics*, vol. 3, no. 2, pp. 209–214, 2016.

[9] X. Sun, S. Huo, H. Yang, M. Yan, J. Zhai, S. Zhao, and Y. Zeng, "Optimizing metasurface-component performance by improving transmittance and phase match of the nanopillars," *Nanomaterials*, vol. 12, no. 21, p. 3720, 2022.

[10] M. Khorasaninejad, W. T. Chen, R. C. Devlin, J. Oh, A. Y. Zhu, and F. Capasso, "Metalenses at visible wavelengths: Diffraction-limited focusing and subwavelength resolution imaging," *Science*, vol. 352, no. 6290, pp. 1190–1194, 2016.

[11] S. Karmakar, A. Ringne, N. Kumar, and A. Krishnan, "Uniform dipole resonance and suppressed quadrupole resonance for constant transmittivity full phase control plasmonic metasurfaces," *Scientific Reports*, vol. 14, no. 1, p. 31499, 2024.

[12] S. Wang, P. C. Wu, V.-C. Su, Y.-C. Lai, M.-K. Chen, H. Y. Kuo, B. H. Chen, Y. H. Chen, T.-T. Huang, J.-H. Wang, *et al.*, "A broadband achromatic metalens in the visible," *Nature nanotechnology*, vol. 13, no. 3, pp. 227–232, 2018.

[13] L. Langguth, A. Schokker, K. Guo, and A. Koenderink, "Plasmonic phase-gradient metasurface for spontaneous emission control," *Physical Review B*, vol. 92, no. 20, p. 205401, 2015.

[14] A. Pors, M. G. Nielsen, and S. I. Bozhevolnyi, "Analog computing using reflective plasmonic metasurfaces," *Nano letters*, vol. 15, no. 1, pp. 791–797, 2015.

[15] E. Bayati, A. Zhan, S. Colburn, M. V. Zhelyeznyakov, and A. Majumdar, "Role of refractive index in metalens performance," *Applied optics*, vol. 58, no. 6, pp. 1460–1466, 2019.

[16] K. E. Chong, L. Wang, I. Staude, A. R. James, J. Dominguez, S. Liu, G. S. Subramania, M. Decker, D. N. Neshev, I. Brener, *et al.*, "Efficient polarization-insensitive complex wavefront control using huygens' metasurfaces based on dielectric resonant meta-atoms," *Acs Photonics*, vol. 3, no. 4, pp. 514–519, 2016.

[17] K. Tanaka, D. Arslan, M. Weissflog, N. Geib, K. Gerold, A. Szeghalmi, M. Ziegler, F. Eilenberger, T. Pertsch, R. Schiek, *et al.*, "Femtosecond pulse shaping with semiconductor huygens' metasurfaces," *Advanced Optical Materials*, vol. 13, no. 16, p. 2500135, 2025.

[18] X. Pan, Y. Deng, Z. Cai, Z. Chen, Y. Ding, Z. Zheng, and F. Ding, "Three-channel wavefront shaping using non-interleaved spin-multiplexed plasmonic metasurfaces," *Advanced Science*, vol. 12, no. 17, p. 2413138, 2025.

[19] Y. Tanhayivash, H. Soofi, and S. Nikmehr, "Phase and amplitude gradient waveguide coupled metasurfaces," *Scientific Reports*, vol. 15, no. 1, p. 19964, 2025.

[20] Y. Saifullah, Q. Chen, G.-M. Yang, A. B. Waqas, and F. Xu, "Dual-band multi-bit programmable reflective metasurface unit cell: design and experiment," *Optics Express*, vol. 29, no. 2, pp. 2658–2668, 2021.

[21] M. Boyarsky, T. Sleasman, M. F. Imani, J. N. Gollub, and D. R. Smith, "Electronically steered metasurface antenna," *Scientific reports*, vol. 11, no. 1, p. 4693, 2021.

[22] D. Rotshild and A. Abramovich, "Wideband reconfigurable entire ku-band metasurface beam-steerable reflector for satellite communications," *IET Microwaves, Antennas & Propagation*, vol. 13, no. 3, pp. 334–339, 2019.

[23] X. Wang, B. Qian, H. Zhao, and H. Chu, "A reconfigurable metasurface with independent manipulation of amplitude and phase," in *2024 IEEE Asia-Pacific Microwave Conference (APMC)*, pp. 459–461, IEEE, 2024.

[24] H. Xue, Z. Lu, X. Ma, Z. Wang, L. Zhu, S. Yue, J. Han, H. Liu, and L. Li, "A reconfigurable metasurface enhancing signal coverage for wireless communication using reduced numbers of pin diodes," *IEEE Transactions on Microwave Theory and Techniques*, vol. 72, no. 3, pp. 1964–1978, 2023.

[25] H. Chen, W.-B. Lu, Z.-G. Liu, and M.-Y. Geng, "Microwave programmable graphene metasurface," *ACS Photonics*, vol. 7, no. 6, pp. 1425–1435, 2020.

[26] C. Huang, J. Liao, C. Ji, J. Peng, L. Yuan, and X. Luo, "Graphene-integrated reconfigurable metasurface for independent manipulation of reflection magnitude and phase," *Advanced Optical Materials*, vol. 9, no. 5, p. 2001950, 2021.

[27] X. Zhuang, W. Zhang, K. Wang, Y. Gu, Y. An, X. Zhang, J. Gu, D. Luo, J. Han, and W. Zhang, "Active terahertz beam steering based on mechanical deformation of liquid crystal elastomer metasurface," *Light: Science & Applications*, vol. 12, no. 1, p. 14, 2023.

[28] M. Zhang, P. Dong, Y. Wang, B. Wang, L. Yang, R. Wu, W. Hou, and J. Zhang, "Tunable terahertz wavefront modulation based on phase change materials embedded in metasurface," *Nanomaterials*, vol. 12, no. 20, p. 3592, 2022.

[29] A. Komar, Z. Fang, J. Bohn, J. Sautter, M. Decker, A. Miroshnichenko, T. Pertsch, I. Brener, Y. S. Kivshar, I. Staude, *et al.*, "Electrically tunable all-dielectric optical metasurfaces based on liquid crystals," *Applied Physics Letters*, vol. 110, no. 7, 2017.

[30] Y. Hu, X. Ou, T. Zeng, J. Lai, J. Zhang, X. Li, X. Luo, L. Li, F. Fan, and H. Duan, "Electrically tunable multifunctional polarization-dependent metasurfaces integrated with liquid crystals in the visible region," *Nano letters*, vol. 21, no. 11, pp. 4554–4562, 2021.

[31] P. Moitra, X. Xu, R. Maruthiyodan Veetil, X. Liang, T. W. Mass, A. I. Kuznetsov, and R. Paniagua-Domínguez, "Electrically tunable reflective metasurfaces with continuous and full-phase modulation for high-efficiency wavefront control at visible frequencies," *Acs Nano*, vol. 17, no. 17, pp. 16952–16959, 2023.

[32] S.-Q. Li, X. Xu, R. Maruthiyodan Veetil, V. Valuckas, R. Paniagua-Domínguez, and A. I. Kuznetsov, "Phase-only transmissive spatial light modulator based on tunable dielectric metasurface," *Science*, vol. 364, no. 6445, pp. 1087–1090, 2019.

[33] Y. Han, S. Chen, C. Ji, X. Liu, Y. Wang, J. Liu, and J. Li, "Reprogrammable optical metasurfaces by electromechanical reconfiguration," *Optics Express*, vol. 29, no. 19, pp. 30751–30760, 2021.

[34] B. Gao, M. Ren, W. Wu, W. Cai, and J. Xu, "Electro-optic lithium niobate metasurfaces," *Science China Physics, Mechanics & Astronomy*, vol. 64, no. 4, p. 240362, 2021.

[35] L. Carletti, A. Zilli, M. Vincenti, M. Finazzi, C. De Angelis, D. Neshev, A. Toma, and M. Celebrano, "Electro-optic lithium niobate metasurfaces for tunable wavefront shaping," in *2022 Sixteenth International Congress on Artificial Materials for Novel Wave Phenomena (Metamaterials)*, pp. X–089, IEEE, 2022.

[36] R. Kanyang, C. Fang, Q. Yang, Y. Shao, G. Han, Y. Liu, and Y. Hao, “Electro-optical modulation in high q metasurface enhanced with liquid crystal integration,” *Nanomaterials*, vol. 12, no. 18, p. 3179, 2022.

[37] D. Barton, M. Lawrence, and J. Dionne, “Wavefront shaping and modulation with resonant electro-optic phase gradient metasurfaces,” *Applied Physics Letters*, vol. 118, no. 7, 2021.

[38] M. C. Sherrott, P. W. Hon, K. T. Fountaine, J. C. Garcia, S. M. Ponti, V. W. Brar, L. A. Sweatlock, and H. A. Atwater, “Experimental demonstration of¿ 230 phase modulation in gate-tunable graphene–gold reconfigurable mid-infrared metasurfaces,” *Nano letters*, vol. 17, no. 5, pp. 3027–3034, 2017.

[39] J. Park, J. Y. Kim, S. Nam, and M. S. Jang, “High-efficiency multi-level beam switching with single-gate tunable metasurfaces based on graphene,” *Advanced Optical Materials*, vol. 13, no. 17, p. 2500236, 2025.

[40] Y.-R. Li, Y. Li, S. Zeng, A. Zhao, S. Yao, M. Zhang, and Z. Li, “Lossless phase-change material enabled wideband high-efficiency spatial light phase modulation at near-infrared,” *Laser & Photonics Reviews*, vol. 18, no. 11, p. 2400293, 2024.

[41] Y. Kim, P. C. Wu, R. Sokhoyan, K. Mauser, R. Glaudell, G. Kafaie Shirmanesh, and H. A. Atwater, “Phase modulation with electrically tunable vanadium dioxide phase-change metasurfaces,” *Nano letters*, vol. 19, no. 6, pp. 3961–3968, 2019.

[42] C. Zhou, Z. Xie, B. Zhang, T. Lei, Z. Li, L. Du, and X. Yuan, “Reconfigurable dielectric metasurface for active wavefront modulation based on a phase-change material metamolecule design,” *Optics Express*, vol. 28, no. 25, pp. 38241–38251, 2020.
